# A MXene Hydrogel‐Based Versatile Microrobot for Controllable Water Pollution Management

**DOI:** 10.1002/advs.202309257

**Published:** 2024-05-05

**Authors:** Kuo Yang, Qianqian Dong, Hang Liu, Lei Wu, Shenfei Zong, Zhuyuan Wang

**Affiliations:** ^1^ Advanced Photonics Center School of Electronic Science and Engineering Southeast University Nanjing 210096 China

**Keywords:** dye pollution, light‐responsive hydrogels, microrobots, MXenes, surface enhanced Raman scattering (SERS)

## Abstract

The urgent demand for addressing dye contaminants in water necessitates the development of microrobots that exhibit remote navigation, rapid removal, and molecular identification capabilities. The progress of microrobot development is currently hindered by the scarcity of multifunctional materials. In this study, a plasmonic MXene hydrogel (PM‐Gel) is synthesized by combining bimetallic nanocubes and Ti_3_C_2_T*_x_* MXene through the rapid gelation of degradable alginate. The hydrogel can efficiently adsorb over 60% of dye contaminants within 2 min, ultimately achieving a removal rate of >90%. Meanwhile, the hydrogel exhibits excellent sensitivity in surface enhanced Raman scattering (SERS) detection, with a limit of detection (LOD) as low as 3.76 am. The properties of the plasmonic hydrogel can be further adjusted for various applications. As a proof‐of‐concept experiment, thermosensitive polymers and superparamagnetic particles are successfully integrated into this hydrogel to construct a versatile, light‐responsive microrobot for dye contaminants. With magnetic and optical actuation, the robot can remotely sample, identify, and remove pollutants in maze‐like channels. Moreover, light‐driven hydrophilic‐hydrophobic switch of the microrobots through photothermal effect can further enhance the adsorption capacity and reduced the dye residue by up to 58%. These findings indicate of a broad application potential in complex real‐world environments.

## Graphical abstract

A strategy for rapid preparation of high‐performance MXene‐based hydrogel and derived versatile microrobots is proposed. The as‐prepared microrobot simultaneously achieves the active control of non‐contact light response and magnetic manipulation, while also retaining high‐level passive capabilities of adsorption and identification (LOD ≈aM, maximum adsorption rate ≈1197 mg g^−1^).

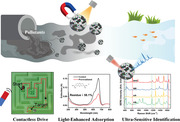

## 1 Introduction

Water pollution poses a significant threat to both human beings and other living organisms. In particular, organic dye contaminants are considered highly hazardous to the environment.^[^ ^1^, ^2^, ^3^ ^]^ This is due to the potential to induce both immediate and long‐term damage, and their contribution to the progression of malignant diseases such as cancer.^[^ ^4^, ^5^, ^6^ ^]^ Thus, the identification and removal of organic dyes from polluted water is a matter of utmost importance for ensuring public health and supporting environmental remediation.^[^ ^7^ ^]^

Recently, researchers have shown significant interest in multifunctional microrobots that can be actuated remotely. This is because optimal sampling sites for pollution management are often indirectly accessible due to factors such as vegetation or terrain.^[^ ^8^ ^]^ Furthermore, in applications such as investigating environmental issues and guiding ecological restoration, advanced robots capable of recognizing and removing dyes simultaneously are urgently needed.

Diverse materials have been prepared and evaluated to address the issue of dye pollutants, including ion exchange resins, activated charcoal (AC), metal‐organic frameworks (MOFs), and functionalized clay.^[^ ^9^, ^10^, ^11^, ^12^ ^]^ These materials have been widely applied because of their simplicity and effectiveness. However, these materials, which only serve the purpose of passive adsorption with a slow adsorption rate, have gradually failed to meet the growing demands of environmental management.^[^ ^13^, ^14^, ^15^, ^16^ ^]^ In addition, the physical or chemical properties of these materials cannot be adjusted after preparation, limiting the versatility of applications. Thus, composite adsorbents possessing scalable functionality have been regarded as attractive candidates for water treatment.^[^ ^17^, ^18^, ^19^, ^20^ ^]^

Most recently, MXenes have attracted particular attention in catalysis, energy, and environmental applications due to their large active surface area and flexible microstructure.^[^ ^21^, ^22^, ^23^ ^]^ Despite the gratifying features of MXenes for molecule adsorption, their practical application is constrained by agglomeration and stacking.^[^ ^24^, ^25^ ^]^ To tackle this problem, porous MXene hydrogels have emerged as a viable solution, offering stable carriers and easily adjustable structures.^[^ ^26^, ^27^, ^28^ ^]^ Advanced studies have shown the potential of MXene hydrogel in environmental applications with improved performance.^[^ ^29^, ^30^, ^31^ ^]^ Additionally, the photothermal effect of Ti_3_C_2_T*_x_* MXene lays the foundation for the development of remotely light‐responsive robots.^[^ ^32^, ^33^ ^]^ Moreover, MXene hydrogels can be employed as SERS sensors in conjunction with noble metal nanoparticles. In our previous research, it was found that the flexible Ti_3_C_2_T*_x_* MXene can enhance the sensitivity of surface enhanced Raman scattering (SERS)‐based sensors due to its strong ability to enrich analytes.^[^ ^34^ ^]^ Thus, the MXene‐based hydrogel may serve as an important tool in the field of environmental analysis, as it can deliver accurate chemical information about pollutants in a non‐contact manner.

Herein, we report a plasmonic MXene hydrogel (PM‐Gel) encapsulating Ti_3_C_2_T*_x_* MXene and AuAgAu nanocubes, which can enable highly efficient adsorption and ultra‐sensitive identification of various organic dye contaminants. The rate of adsorption rate and final removal rate far exceed that of commercially available AC. After just 2 min of exposure, the hydrogel was able to adsorb over 60% of typical dyes such as methylene blue (MB), resulting in a final removal rate of over 90%. In terms of the mass of doped MXene, the maximum adsorption capacity reached 1197.00 mg g^−1^. Besides, the hydrogel‐based SERS sensor demonstrated exceptional sensitivity to trace contaminations. The LOD for the cationic dye rhodamine 6G (R6G) was as low as 3.76 am. Additionally, the PM‐gel also exhibited a high sensitivity to anionic dyes, with an LOD of 16.33 fm for acid red (AR).

Furthermore, a versatile light‐responsive robot based on the PM‐gel was developed for the treatment of dye contaminants. Thermosensitive polymers and γ‐Fe_2_O_3_ nanoparticles were incorporated into alginate frameworks through chemical and physical interactions, providing the robot with multiple functions. The controlled sampling, identification, and removal of dyes can all be achieved through contactless magnetic or light manipulation. As a proof‐of‐concept experiment, the operation of microrobots in maze‐like channels was proposed, indicating a high potential for practical applications in complex real environments. Additionally, we also demonstrated that the adsorption capacity can be further enhanced by a light‐driven hydrophilic–hydrophobic switch through the photothermal effect. This effect notably reduced the amount of dye residue by 58.1%.

## 2 Results and Discussion

### 2.1 Fabrication and Characterization of the Plasmonic MXene Hydrogel

**Figure 1:**
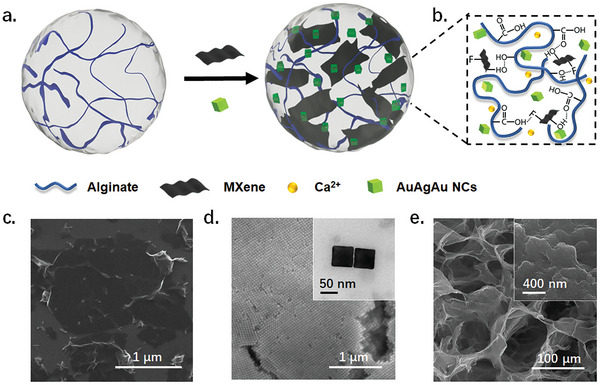
Preparation and characterization of the PM‐Gel. a) The schematic preparation route of the hydrogel. b) The schematic diagram of the cross‐linked structure of hydrogel. c) The SEM image of Ti_3_C_2_T*_x_* MXene. d) The SEM image of AuAgAu nanocubes. The inset image is the TEM image of nanocubes. e) The SEM image of the freeze‐dried PM‐Gel. The inset image is the high‐magnification SEM image of the PM‐Gel.

To rapidly prepare the composite hydrogels, we utilized eco‐friendly Na–Alg as the primary hydrogel matrix. The viscous Na–Alg solution containing flexible Ti_3_C_2_T*_x_* MXene and AuAgAu nanocubes was injected dropwise into a calcium chloride (CaCl_2_) solution controlled by a syringe pump, resulting in the formation of PM‐Gel (**Figure** **1**a). Then, the Ca^2+^ accomplished the cross‐linking of the hydrogel in the following 5 minutes. During this process, the abundant terminations of MXene, the surface ligands of the nanoparticles, and the coordinating groups of polymers all contributed to the formation of the hydrogel (Figure 1b).

The MXene nanosheets were examined using electron microscopy, confirming their optimal morphology and size. As shown in Figure 1c and Figure S1 (Supporting Information), it can be observed that the MXene displays a wrinkled morphology, suggesting that the Ti_3_C_2_T*_x_* nanosheets are primarily composed of a few layers or monolayer in structure. Besides, the peak observed at 39° in the XRD pattern nearly vanished, suggesting the selective removal of the aluminum layer. In addition, the (002) peak at 2*θ* = 9.6° was observed to shift by 7.1°, proving evidence for the effective exfoliation of Ti_3_C_2_T_*x*_ nanosheets (Figure S2, Supporting Information). The AuAgAu NCs were also subjected to characterization. The monodispersibility of the samples was confirmed by the SEM and TEM images (Figure 1d; Figure S3, Supporting Information). During the nanoparticle growth process, nanoparticles with a cubic shape exhibit distinct surface plasmon resonance (SPR) peaks below 400 nm, which are associated with dipole resonance modes (Figure S4, Supporting Information). The typical EDX elemental maps provide additional clarification on the distribution of Au and Ag, confirming the onion‐like structure (Figure S5, Supporting Information). Additionally, the SEM images revealed the microscopic roughness of the hydrogel matrix (Figure 1e and Figure S6, Supporting Information), showcasing pores ranging from 100–200 µm. This characterizes a feature that facilitating swift liquid exchange.

**Figure 2:**
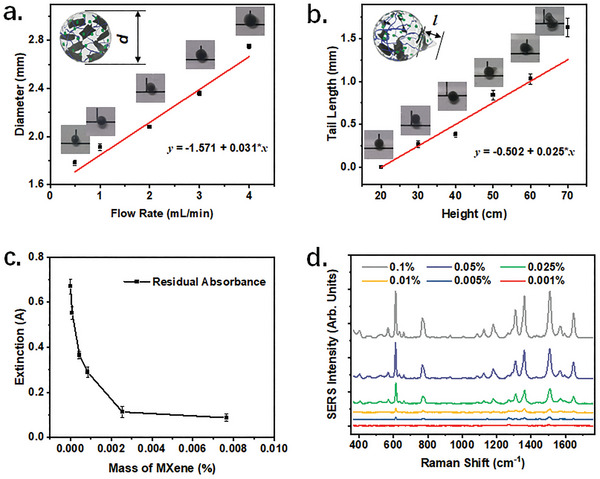
Varied properties of the PM‐Gel by different gelation parameters. a) Changes in the diameter of spherical hydrogels with the flow rate of precursor solutions. b) Changes in the tail length of deformed spherical hydrogels with the drop height of precursor solutions. c) The residual absorbance of the R6G solutions (5 mL, 10^−5^ m) at 526 nm after being incubated with the hydrogels containing different amounts of Ti_3_C_2_T*_x_* MXene. d) The SERS spectra of the R6G (10^−8^ m) acquired by the PM‐Gel containing different amounts of bimetallic nanocubes. Data are presented as mean ± standard error of the mean, n = 5.

By adjusting the preparation parameters, it is possible to modify the morphology and functionality of the plasmonic MXene hydrogel. For instance, elevating the injection rate from 0.5 to 4 mL min^−1^ resulted in a consistent increase in the size of hydrogel beads (**Figure** **2**a). As the height difference between the precursor outlet and the CaCl_2_ pool increases, the hydrogel displays a trailing effect due to the higher final velocity (Figure 2b). Meanwhile, the modulation of hydrogel morphology hardly effects its sensing performance (Figure S7, Supporting Information). As indicated in the recent report by Zhao et al., alginate hydrogels can be even transformed into multicore fibers.^[^ ^35^ ^]^ Adjusting the concentration of individual components could modulate the properties of the hydrogel as well. Specifically, the augmentation of MXene content has been found to be advantageous in improving adsorption capacity (Figure 2c), whereas the augmentation of nanoparticle content has been observed to enhance SERS detection (Figure 2d). Given the gradual saturation of positive effects, the concentrations of MXene and nanoparticles were optimized as 0.0025% and 0.05% for subsequent experiments, respectively. Besides, the high concentrations indicate favorable loading capacity and stability of the hydrogel.

### 2.2 High‐Efficient Dye Removal by the Plasmonic MXene Hydrogel

The adsorption capacity of the hydrogel towards various dyes was investigated. The ultimate rate of the plasmonic MXene hydrogel significantly exceeded that of commercially available AC. Additionally, the data confirmed that Ti_3_C_2_T*_x_* MXene served as the primary absorbent within the hydrogel. In the experiments, R6G and MB were selected as the typical cationic dyes, while acid red (AR) and acid blue (AB) were chosen as examples of anionic dyes.

Figures S8–11 (Supporting Information) display the absorbance calibration curves of different dyes. The linearity of the standard curve was observed for each dye within the concentration range of 0.5 to 20 µm. The PM‐Gel beads were subsequently introduced into the dye solutions (10 µm). After a specific timeframe, the dye adsorption rate was determined by monitoring the absorbance of the supernatant.

**Figure 3:**
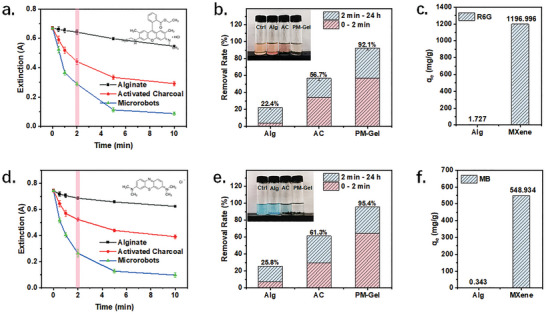
Investigation of the dye adsorption ability of the PM‐gel. a) The variation of absorbance at 526 nm of R6G solution (10 µm) that incubated with Alg hydrogel, AC, and PM‐Gel for different time. The pink bar marks the data recorded at 2 min. b) The removal rates of R6G (10 µm) by different materials. The red part represents of the removal rate at 2 min, while the blue part represents the changes in the removal rate for the remaining time. The inset photo shows the R6G solutions after 24 h incubation. c) Adsorption capacity of different materials for R6G. d) The variation of the absorbance at 662 nm of MB solution (10 µm) incubated with Alg hydrogel, AC, and PM‐Gel for different time. The pink bar marks the data recorded at 2 min. e) The removal rates of MB (10 µm) by different materials. The red part indicates the removal rate at 2 min, while the blue one indicates the changes of removal rate for the remaining time. The inset photo shows the MB solutions after 24 h of incubation. f) Adsorption capacity of different materials for MB. Data are presented as mean ± standard error of the mean, *n* = 5.

The MXene hydrogel exhibited a significantly higher removal rate in comparison to AC and pure alginate (Alg) hydrogel. The hydrogel exhibits a capability to eliminate ≈56.9% of R6G and 64.3% of MB within only 2 min. This rate of removal is double that achieved by commercially available AC (**Figure** **3**a,d). The dyes exhibited a maximum removal rate of 95% within 2 hours, as depicted in Figure 3b,e. In addition, the hydrogel displayed no release of debris into the solution, thereby potentially facilitating subsequent recycling processes. Conversely, it is observable from the images that the solution with AC darkened after shaking, as a result of the suspension of charcoal debris in the water.

Besides, the adsorption behavior of the materials was subjected to kinetic analysis (Figure S12 and Table S13, Supporting Information). The adsorption model was identified as pseudo‐second‐order adsorption. The saturated adsorption rates of MXene on R6G and MB were estimated to be 1197.00 and 548.93 mg/g, respectively (Figure 3c,f). Meanwhile, the value of pure Alg hydrogel was only 1.73 mg/g. The results clearly demonstrate that MXene serves as the primary absorbent in the composite hydrogel (Figure S14, Supporting Information). For anionic dyes, the PM‐Gel also showed a notable adsorption capacity, as illustrated in Figure S15 (Supporting Information).

Meanwhile, the hydrogel exhibits a significantly higher efficiency in comparison to conventional adsorbents such as AC, which has a saturation capacity of approximately 7% in alkaline dye solutions of R6G and MB.

Overall, the proposed PM‐Gel exhibits a significant potential as an efficient dye scavenger, whose performance is even better than that of AC.

### 2.3 Ultra‐Sensitive Identification of Multiple Dyes by the Plasmonic MXene Hydrogel

Traditional adsorbent materials usually possess singular functionality. Consequently, they are unable to offer additional details regarding pollutants. This leads to the need for time‐consuming post‐processing procedures to separate pollutants for further detection. As a result, the efficient monitoring and management of water pollutants is thereby hindered, causing delays in assessing their ecological impact. In this work, the fingerprint spectra of dyes can be readout directly by the SERS‐active PM‐Gel, without further process.

**Figure 4:**
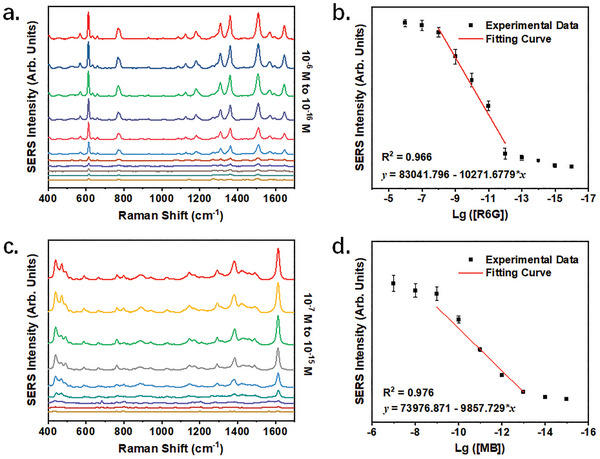
Investigation of the SERS activity of the PM‐Gel. Detection results of a) R6G, and c) MB at different concentrations by the PM‐Gel. Before tests, the hydrogel beads were immersed in the dye solutions at 10 µM and then washed by pure water. SERS intensity of b) R6G, and d) MB at different concentrations based on the peak at 612 or 1613 cm^–1^, respectively. Data are presented as mean ± standard error of the mean, n = 5.

The SERS activity of the plasmonic hydrogel was assessed by employing a typical dye of R6G. In the experiments, the hydrogel beads were immersed in R6G solutions of varying concentrations prior to detection. As shown in **Figure** **4**a, an extraordinarily low LOD of 3.76 aM was achieved (calculated by the 3N method), demonstrating an exceptionally high level of sensitivity. The average enhancement factor (AEF) for R6G was calculated to be 5.5×10^6^ (Figure S16, Supporting Information). Considering the relative low AEF, the remarkable sensitivity should be attributed to the effective enrichment capability of the MXene hydrogel. Meanwhile, the sensor demonstrated a linear range as wide as 5 orders of magnitude (Figure 4b). The signal fluctuation among multiple hydrogel beads, based on the peak intensity at 612 cm^−1^, was measured to be 11.1% (Figure S17, Supporting Information). These results indicate a high level of homogeneity among the beads.

Additionally, we evaluated the SERS activity of the PM‐Gel for the other three kinds of dyes. Similarly, the PM‐Gel demonstrated a remarkable sensitivity to MB, achieving a LOD as low as 1.98 fm (Figure 4c,d). In addition, the detection performance for anionic dyes was deemed satisfactory, as evidenced by LOD values of 16.33 fm and 381.07 pm for AR and AB, respectively (Figure S18, Supporting Information).

**Figure 5:**
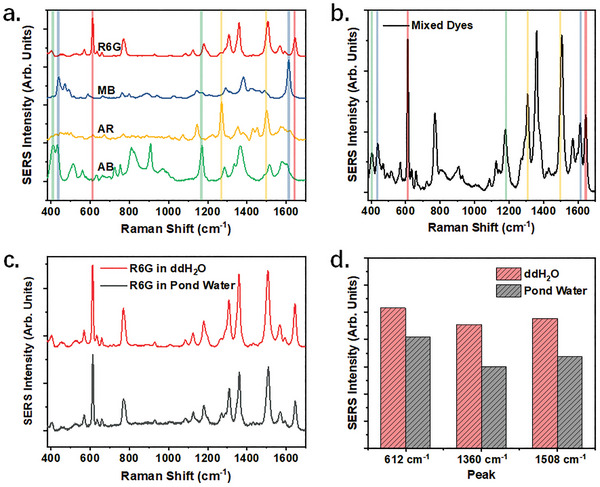
Investigation of the multiplex detection capability of the PM‐Gel. a) Detection results of R6G, MB, AR, and AB at 1 µm. b) Detection results of the mixture containing R6G, MB, AR, and AB. The concentrations of four kinds of dyes are 1 µm. The characteristic peaks at 612 cm^−1^ and 1644 cm^−1^ are used for identifying R6G, the peaks at 438 cm^−1^and 1613 cm^−1^ are used for identifying MB, the peaks at 1270 cm^−1^ and 1499 cm^−1^ are used for identifying AR, and the peaks at 411 cm^−1^ and 1168 cm^−1^ are used for identifying AB. c) Spectra of R6G detected in ultrapure water and natural pond water. The concentration of the spiked R6G is 1 µm. d) The corresponding SERS intensity in (c) at 612 cm^–1^, 1360 cm^–1^, and 1508 cm^–1^, respectively. Data are presented as mean ± standard error of the mean, n = 5.

Furthermore, the utilization of SERS signal endowed the PM‐Gel with the capability for multiplex detection, thereby holding significant implications for its practical applications. In the experiments, four dyes could be identified simultaneously from a single spectrum of mixture solutions by assigning distinct characteristic SERS peaks (**Figure** **5**a,b).

Moreover, an investigation into the stability of hydrogels was conducted. The performance of PM‐Gel was maintained well across a wide pH (5–10) and temperature ranges (10–30 °C; Figure S19, Supporting Information). Besides, the decrease of adsorption capacity was determined less than 10% after a 20‐day storage, showing favorable long‐term stability (Figure S20, Supporting Information). In addition, the signal error due to incomplete desorption was identified to be ≈20% after five cycles (Figure S21, Supporting Information). Meanwhile, no dissolution and release of nanoparticles and MXenes in solution were observed during the experiments.

Besides, the PM‐Gel exhibits a significant advantage due to its large pore size and abundant adsorption sites, enabling it to effectively withstand external disturbances caused by suspended impurities present in specimens. As depicted in Figure 5c, the distortion of the spectrum and the notable decrease in SERS intensity indicated of the presence of impurities in pond water, which disrupted the detection process. However, upon comparing the two spectra presented in Figure 5c, it is evident that the distinctive peaks remain prominent, thereby indicating the sustained specificity in the identification of impure pond water (Figure 5d).

Overall, the PM‐Gel presents notable enhancements in sensing capabilities when compared to previous approaches.^[^ ^36^, ^37^ ^]^ The excellent stability also supports its potential applications in the field of environmental monitoring.

### 2.4 Microrobots for Controllable Sampling and Detection of Dye Contaminants in Inaccessible Areas

**Figure 6:**
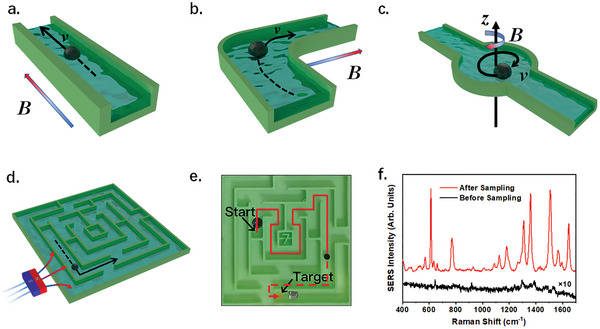
Controllable sampling and detection of dye contaminants. The navigation schematic diagrams of a) forward movement, b) turning, and c) spinning. ***B*** represents the magnetic flux density. d) The maze‐like microfluidic channels mimicking the inaccessible areas in real applications. e) The screen shot of Video S1 (Supporting Information). The red line marks the movement trajectory of the microrobot. f) SERS results of the microrobot before and after the controlled sampling of R6G (0.1 µL, 10 µm) in the maze‐like microchannels.

In practical applications involving pollutant monitoring, the availability of manual direct sampling is often limited due to factors such as terrain, vegetation, and security issues. In this particular scenario, the significance of remotely controlled sampling and contamination removal cannot be overstated. By incorporating superparamagnetic materials into hydrogels, the PM‐Gel has the capability to transform into magnetic microrobots. These microrobots can be precisely controlled to execute straight, turning, and spinning motions, enabling effective sampling, detection, and elimination dye contaminants in complex and intricate areas (**Figure** **6**a–c and Figures S22 and S23, Supporting Information).

Herein, uniform γ‐Fe_2_O_3_ nanoparticles were employed as the superparamagnetic materials (Figures S24 and S25, Supporting Information). These nanoparticles possessed a saturation magnetic moment of 40 emu g^−1^ (Figure S26, Supporting Information). The desirable superparamagnetic properties avert the aggregation of nanoparticles and the collapse of the hydrogel.

As a feasibility test for sampling purposes, we designed a microfluidic channel with a maze‐like structure to demonstrate the microrobot's ability to navigate and remove pollutants in a challenging application environment (Figure 6d). We assumed that the walls in the diagram represent the barriers that exist between the accessible point and inaccessible target sampling point, which were denoted as “Start” and “Target”, respectively (Figure 6e). The process comprises several stages, including targeting, adsorption, homing, and detection. Initially, a superparamagnetic microrobot was placed at the designated “Start” location. Subsequently, it was actuated to navigate through the zigzag channel until reaching the designated “Target” point. During the period, the navigation of the robot was calibrated by means of an external magnet (Video S1, Supporting Information). To optimize the process of dye sampling and adsorption, the robot was programmed to rotate at the “Target” point. After a certain period of time, the robot retraced its path and the inclusions could be meticulously examined through SERS detection. As demonstrated in Video S1 (Supporting Information), the proposed microrobot efficiently sampled and eliminated contamination consisting of a 0.1 µL droplet of R6G (10 µm) within a few seconds. Afterward, the chemical information of the indirectly accessible contaminants can be determined through analysis of the returning microrobot (Figure 6f).

### 2.5 Light‐Enhanced Adsorption of Dyes by Microrobots Based on Photothermal Effect

**Figure 7:**
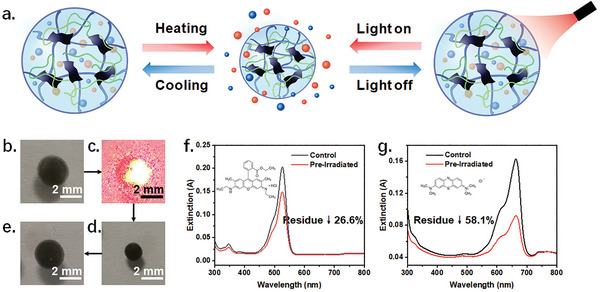
Light‐induced enhancement of dye adsorption. a) The schematic diagram of light‐ or temperature‐controlled reversible shrinkage of the microrobot. The colored spheres in the image represent the presence of dye contaminants. Photographs of a microrobot b) in original state, c) under laser irradiation, d) shrunk to minimum, and e) after expansion recovery. f–g) Comparison of the ability to remove the same dye (300 µL, 10 µm) in 5 min using a light‐shrunk microrobot and an untreated one. Data are presented as mean ± standard error of the mean, *n* = 5.

The utilization of the photothermal effect of Ti_3_C_2_T*_x_* MXene allows for a contactless heating approach, facilitating remote manipulation of temperature‐sensitive hydrogels that incorporate *N*‐isopropyl acrylamide (NIPAM).^[^ ^38^, ^39^ ^]^ By subjecting the MXene hydrogel‐based microrobots doped with NIPAM to laser irradiation, they underwent shrinkage in response to temperature changes, leading to the adoption of a hydrophobic state (Figure S27, Supporting Information). When the laser excitation was absent or the temperature was reduced, the microrobots would restore their initial configuration (**Figure** **7**a). The typical transition temperature was found to be 32°C.^[^ ^40^ ^]^ In experiments, the microrobot underwent a volume shrinkage of up to 82.2%, with a subsequent recovery rate of 86.6% (Figure 7b–d). During this process, the surrounding solution should migrate rapidly as the state of microrobots changed. The induced mass transfer probably enhances the fixation of dye contaminants.

In the conducted experiment, it was observed that the shrunken microrobots, which had been pre‐irradiated, demonstrated a notable improvement in dye removal capacity. In comparison to the control group, the experimental group exhibited a 26% decrease in dye residue for R6G and a 58% decrease for MB, as depicted in Figure 7f,g, respectively. Such beneficial effect should be attributed to the enhanced mass transfer triggered by light‐driven hydrophilic‐hydrophobic switch. This also presents a potential avenue for the optimize other porous and flexible materials.

## 3 Conclusion

In conclusion, a versatile PM‐Gel microrobot was successfully constructed. The composite hydrogel exhibited remarkable efficacy in the removal and identification of dyes, facilitated by the incorporation of different colloids such as Ti_3_C_2_T*_x_* MXene and AuAgAu NCs. The removal rate exceeded 90%, whereas the adsorption rate within 2 min was double that of AC. In addition, it attained remarkably low LODs of 3.76 am for cationic dyes and 16.33 fm for anionic dyes. The study also involved conducting remote dye pollution management, wherein microrobots were able to effectively target, sample, and identify contaminants that were otherwise inaccessible. Furthermore, the light‐driven augmented adsorption of the hydrogel robot was investigated as well. Compared with previously reported works (Table S28, Supporting Information), the PM‐Gel exhibits superior performance in terms of sensitivity, saturation adsorption rate, versatility, and scalability. Besides, the proposed composite materials are well biocompatible and thus do not pose an additional environmental burden in applications. We believe that such a kind of robot has the potential to contribute to environmental conservation. Moreover, there is great promise in the development of new technological methods for various applications, including vascular dredging, drug delivery, and chemical reaction monitoring. However, there are still many challenges. For instance, in the case of adsorbent materials for widespread application, it is crucial to improve that the preparation process of expensive MXenes. Additionally, the accuracy of light‐driven manipulation needs to be improved.

## 4 Experimental Section

### Materials

Sodium alginate (Na–Alg), calcium chloride (CaCl_2_), rhodamine 6G (R6G), methylene blue (MB), acid blue 93 (AB), acid red 27 (AR), oleic acid (90%), 1‐octadecene (90%), silver nitrate (AgNO_3_, ≥ 99.999%), ascorbic acid (AA), and lithium phenyl‐2,4,6‐trimethylbenzoylphosphinate (LAP, 95%) were purchased from Sigma–Aldrich. Sodium oleate (NaOA) was purchased from TCI. Hydrogen tetrachloroaurate trihydrate (HAuCl_4_·3H_2_O ≥ 99.995%), lithium fluoride (LiF), ferric chloride (FeCl_3_·6H_2_O), hexadecyl trimethylammonium bromide (CTAB), and hexadecyl trimethyl ammonium chloride (CTAC) were purchased from Alfa Aesar. Ethanol, hexane, bis‐acrylamide (BIS), and *N*‐isopropyl acrylamide (NIPAM) were purchased from Sinopharm. Activated charcoal (AC) was purchased from Honeywell. Distilled water (Millipore Milli‐Q grade) with a resistivity of 18.2 MΩ cm^−1^ was used in all the experiments. Ti_3_AlC_2_ was purchased from Laizhou Kai Kai Ceramic Materials. The pond water was sampled in the Xuanwu Lake. All the reagents and materials were used as received.

### Preparation of the Nanomaterials

Solutions of MXene were prepared by a freeze–thaw method.^[^ ^41^ ^]^ Nanocubes with Au–Ag–Au structure were prepared by a seed growth method.^[^ ^42^ ^]^

Superparamagnetic nanoparticles with a diameter of 14 nm were prepared by thermal decomposition method.^[^ ^43^ ^]^ Briefly, FeCl_3_·6H_2_O (10.8 g) and NaOA (36.5 g) were dissolved in a mixed solution of water (60 mL), ethanol (80 mL), and n‐hexane (140 mL) at 70 °C. The mixture was then refluxed for 3 h. The products were then extracted by a separatory funnel and washed with water for three times. Finally, the waxy precursor of iron–oleate complex was obtained by vacuum drying of 6 h. After that, the precursor (3.6 g) and oleic acid (0.57 g) were dissolved in 1‐octadecene (20 g) at room temperature. Then the solution was subsequently heated to 330 °C, and kept for 30 min. Afterward, ethanol was added to precipitate the nanocrystals. The nanocrystals were redispersed in 1 m TMAOH/BtOH with the help of ultrasound treatment (SB4200D, Ningbo Scientz Biotechnology). Finally, the superparamagnetic nanoparticles were washed with water and ethanol three times and stored in water.

### Fabrication of the PM‐Gel and the Derived Microrobot

The plasmonic MXene hydrogel was prepared as the following methods. A solution containing MXene (0.0025 wt%), Na‐Alg (1 wt%), and nanocubes (0.05 wt%) was injected dropwise into CaCl_2_ solution (2 wt%) by a syringe pump (flow rate: 1 mL min^−1^, drop height: 20 cm, the inner/out diameter of the flat nozzle: 0.5 mm/0.9 mm) to form the sphere bead of plasmonic MXene hydrogel. The shape of the hydrogel was controlled by adjusting the injection speed and other gelation conditions.

For preparing spherical microrobots with a diameter of 1.75 mm, a solution containing MXene (0.0025 wt%), Na‐Alg (1 wt%), nanocubes (0.05 wt%) superparamagnetic nanoparticles (0.1 wt%), NIPAM (7.5 wt%), BIS (0.25 wt%), and LAP (0.01 wt%) were injected as above. Then, the robots were exposed to UV light for 30 s (3 W cm^−2^), kept in CaCl_2_ solution for 5 min, and washed three times. The microrobots were stored in water before use. NIAPM, BIS, and LAP were only used for light‐driven applications and so was the UV exposure.

### Measurement of the Extinction Spectra

The PM‐Gel, pure alginate hydrogel, or commercial AC of 5 mg (dry weight) were placed in different dye solutions (5 mL, 10 µm), which were allowed to stand for different times. Experiments in this study were all performed at 20 °C and the dye solutions used were neutral. Then the supernatant was taken out for extinction spectroscopy.

### SERS Detection of Dyes Using PM‐Gel

0.25 mg of PM‐Gel (dry weight) were placed in 2 mL of the dye solutions at different concentrations. The solutions were allowed to stand for 2 h. The as‐prepared robot was then taken out and a filter paper was used to remove excess liquid. In Raman detection, the laser was focused on the surface of the microrobot. Before analysis, all SERS spectra were subjected to baseline subtraction and smoothing using LabSpec5 software (Horiba Scientific). The intensity of the most prominent characteristic peak was used as the overall intensity of dye spectra. Specifically, R6G refers to the peak intensity at 612 cm^−1^, MB refers to the peak intensity at 1613 cm^−1^, AR refers to the peak intensity at 1270 cm^−1^, and AB refers to the peak intensity at 1168 cm^−1^. Five random tests were performed for each sample.

### Controllable Navigation of the Microrobot in Maze‐Like Microfluidic Channels

The geometry of the maze‐like microfluidic channels is shown in Figure S29 (Supporting Information). The overall size of the channels was 40×40×3 mm. The width of a channel was 5 mm and the height was 3 mm. Before depositing a microrobot at the “Start” point, the channels were fueled with pure water. The movement of the microrobot was controlled by a NdFeB magnet. The magnetic flux density in the channel was ≈0.1 T.

### Light‐Enhanced Adsorption of Dyes by Microrobots

A microrobot was first located in a perish dish fueled with pure water. Then a laser generated by a laser diode (Sharp, GH0631IA2G) was focused on the microrobot (power: 180 mW, central wavelength: 638 nm, spot size: ≈1 mm in radius) for 5 min. Along with the irradiation, the microrobot gradually shrunk. After that, the microrobot was transferred to another Petri dish containing dye solutions (0.5 mL, 10 µm) and left for 2 h. Finally, the residual absorbance was recorded by a spectrometer.

### Instruments

SEM images were obtained with an FEI Inspect F50 or a Nova NanoSEM 450. TEM images were obtained with a FEI Tecnai G2T20 electron microscope. Digital photos were taken by a Huawei P30 Pro. Syringe pumps (Pump 11 Pico Plus Elite, Harvard Apparatus Ltd.) were used in the microfluidic operation. SERS measurements were performed by a 10× objective lens using duoscan mode at the excitation of 632.8 nm on a Horiba T64000 Raman system. The laser power was 2 mW at the sample position. Each SERS spectrum was an accumulation result of 5 s. The extinction spectra of nanoparticles were measured by a Shimadzu UV‐3600 UV–Vis‐NIR spectrophotometer.

### Statistical Analysis

Analyzed number (n) of samples and repeat times were listed for each experiment as described above. Quantitative data were presented in the figures as mean ± standard deviation of the mean (SD). Except the pre‐processing of Raman spectra, all statistical analyses of spectra data were performed using the OriginLab software.

## Conflict of Interest

The authors declare no conflict of interest.

## Supporting information

Supporting Information

Supplemental Video 1

## Data Availability

The data that support the findings of this study are available from the corresponding author upon reasonable request.
