# Supplementary material for: A MXene Hydrogel‐Based Versatile Microrobot for Controllable Water Pollution Management
Source: Adv Sci (Weinh). 2024 May 5;11(26):2309257. doi: 10.1002/advs.202309257 (PMC11234425; doi:10.1002/advs.202309257)
Supplement: Supplementary file 1 — Supporting Information [file ADVS-11-2309257-s002.pdf]

ADVANCED SCIENCE
Open Access

Supporting Information

for *Adv. Sci.*, DOI 10.1002/advs.202309257

A MXene Hydrogel-Based Versatile Microrobot for Controllable Water Pollution Management

*Kuo Yang, Qianqian Dong, Hang Liu, Lei Wu, Shenfei Zong and Zhuyuan Wang**

Supporting Information

A MXene Hydrogel-Based Versatile Microrobot for Controllable Water Pollution Management

Kuo Yang #, Qianqian Dong #, Hang Liu, Lei Wu, Shenfei Zong, and Zhuyuan Wang *

Advanced Photonics Center, School of Electronic Science and Engineering, Southeast University, Nanjing 210096, China

#These authors contributed equally.

*Email: wangzy@seu.edu.cn

Supplementary Materials List

Figure S1. The typical TEM images of $Ti_3C_2T_x$ MXene.

Figure S2. The XRD patterns of Ti_3AlC_2 and $Ti_3C_2T_x$ MXene.

Figure S3. The SEM image of nanocubes.

Figure S4. Extinction spectra of AgNPs, AuAg nanocubes, and AuAgAu nanocubes.

Figure S5. HAADF-TEM image and EDX elemental mapping of AuAgAu nanocubes.

Figure S6. The typical SEM images of the freeze-dried PM-Gel.

Figure S7. The plasmonic MXene hydrogel of varied shapes.

Figure S8-11. The extinction data of standard dye solutions of different concentrations.

Figure S12. Kinetic modelling analysis for Alg and PM-Gel.

Table S13. Overview of the Kinetic Parameters

Figure S14. Extinction spectra of cationic dye solutions incubated with different materials.

Figure S15. Extinction spectra of anionic solutions incubated with the microrobot.

Figure S16. The calculation of average enhancement factor.

Figure S17. The spectra of R6G (1 μM) detected by different microrobots.

Figure S18. Investigation of the SERS activity of the microrobot for anionic dyes.

Figure S19. Changes in SERS intensity acquired by the PM-Gel under different pH values and temperature.

Figure S20. Changes in SERS intensity acquired by the PM-Gel after 20 days of storage.

Figure S21. Reusability of the PM-Gel.

Figure S22. The structure diagram of a spherical microrobot.

Figure S23. The typical SEM images of the freeze-dried spherical microrobot.

Figure S24. The TEM image of γ-Fe_2O_3 nanoparticles.

Figure S25. The XRD spectra of the γ-Fe_2O_3 nanoparticles.

Figure S26. The hysteresis curves of γ-Fe_2O_3 nanoparticles.

Figure S27. The changes in contact angles of the PM-Gel.

Table S28. Overall features of our work in comparison with other typical materials.

Figure S29. The plane view of the maze-like microfluidic channels.

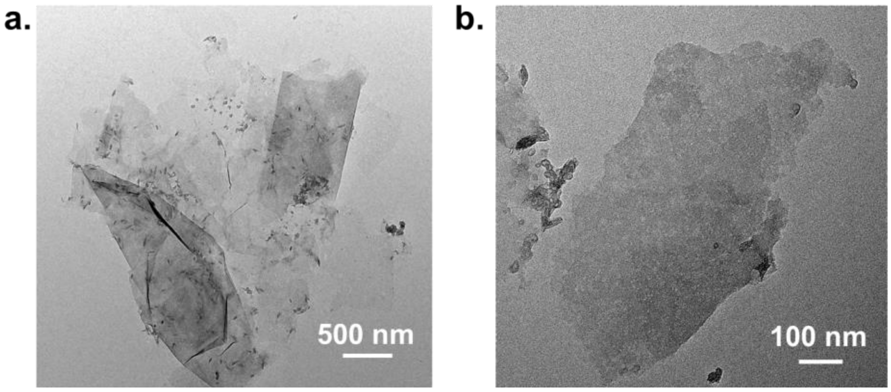

Figure S1. The typical TEM images of $Ti_3C_2T_x$ MXene.

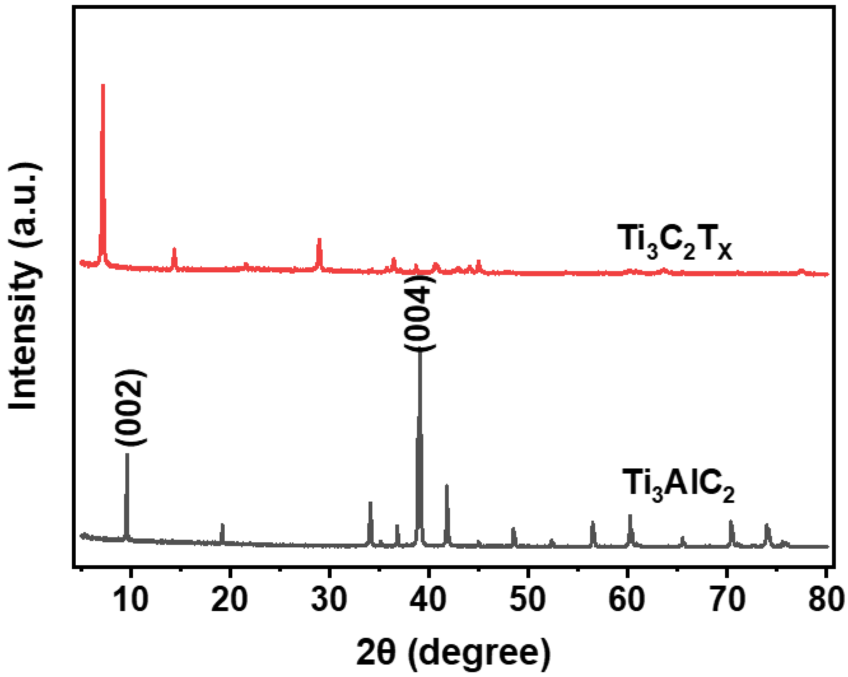

Figure S2. The XRD patterns of Ti_3AlC_2 and $Ti_3C_2T_x$ MXene.

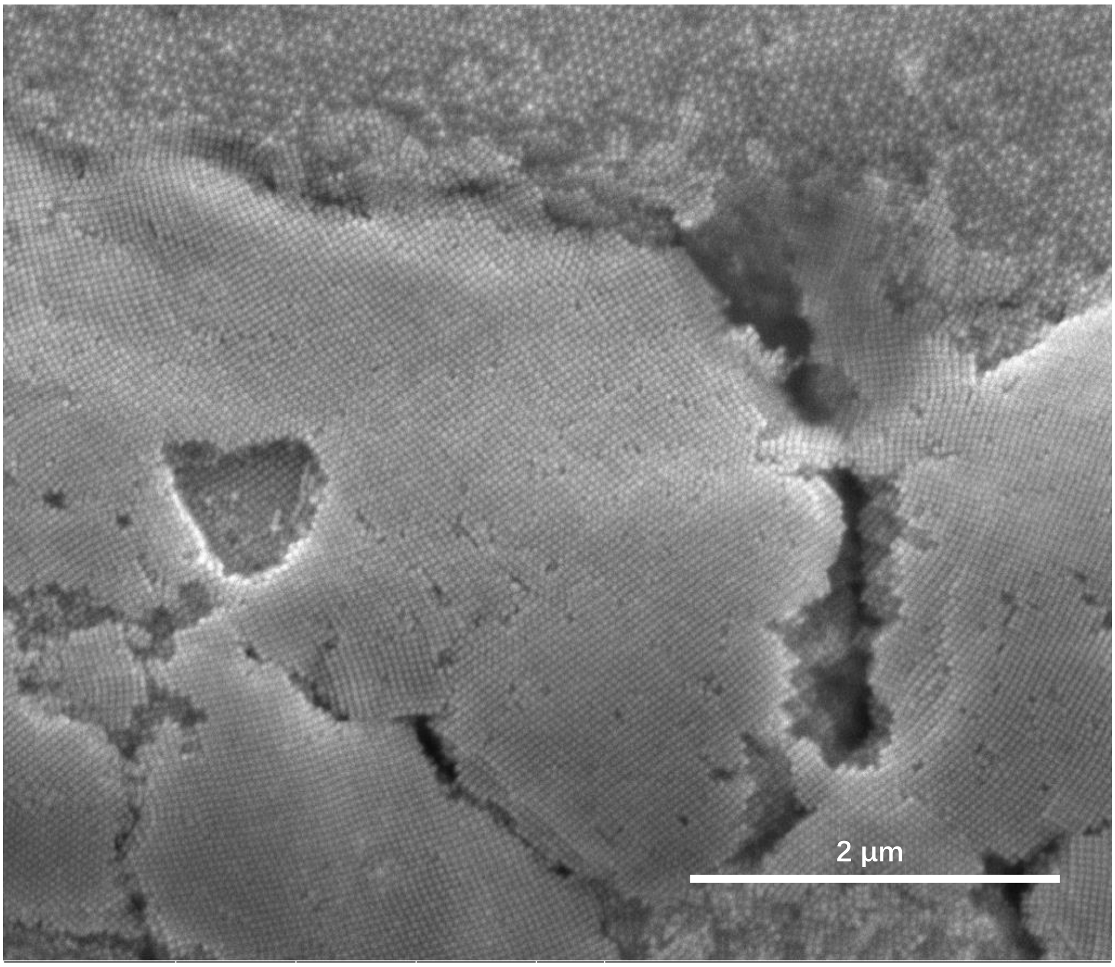

Figure S3. The SEM image of nanocubes.

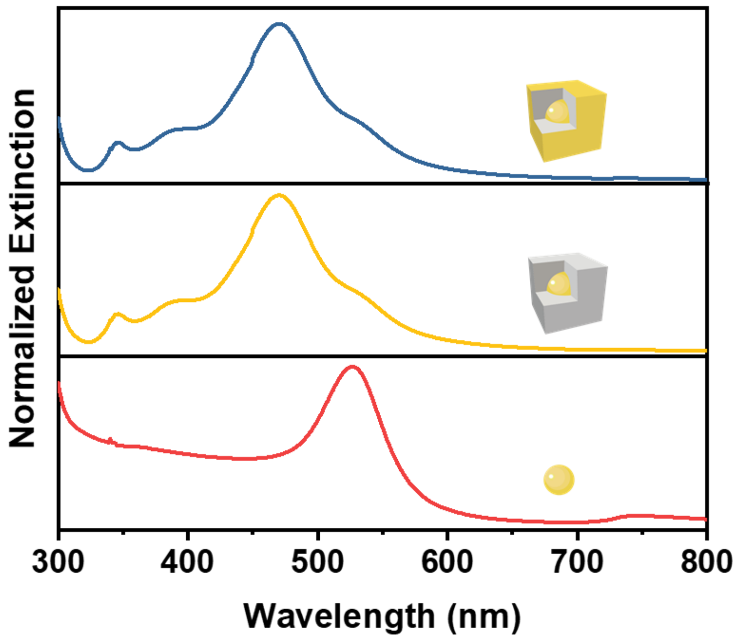

Figure S4. Extinction spectra of AgNPs, AuAg nanocubes, and AuAgAu nanocubes.

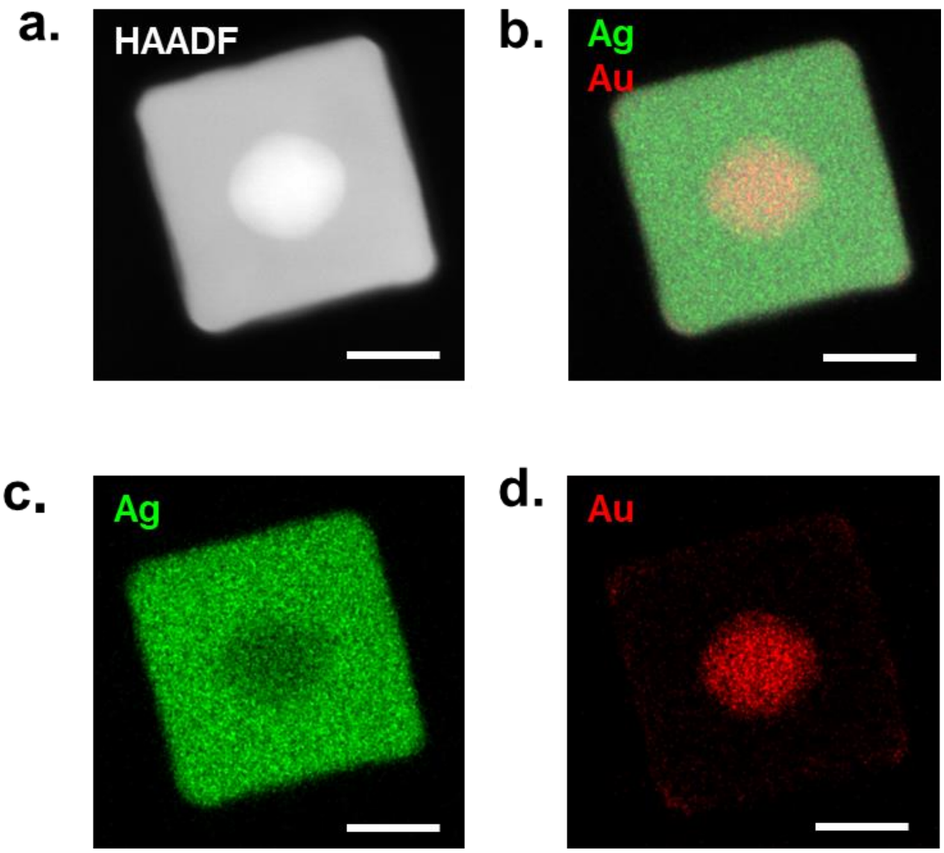

Figure S5. (a) The typical HAADF-TEM image and (b) EDX elemental mapping of AuAgAu nanocubes. (c-d) The typical elemental mapping results of Ag and Au, respectively. The scale bars are all 20 nm.

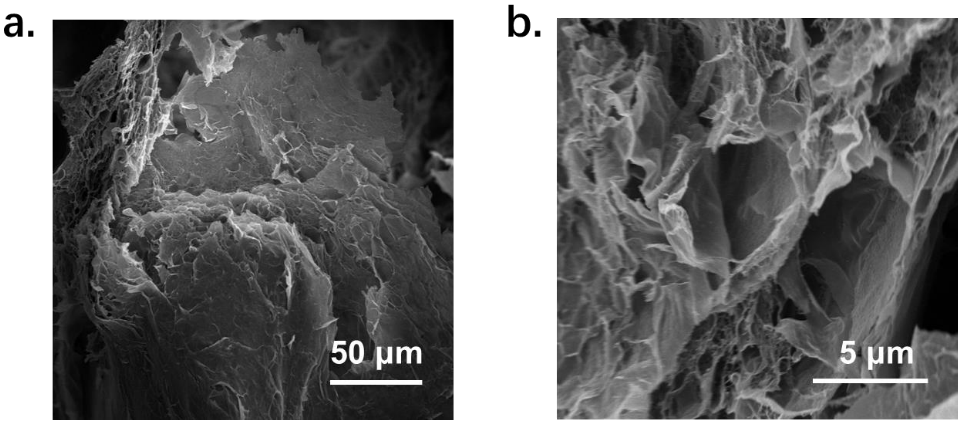

Figure S6. The typical SEM images of the freeze-dried PM-Gel.

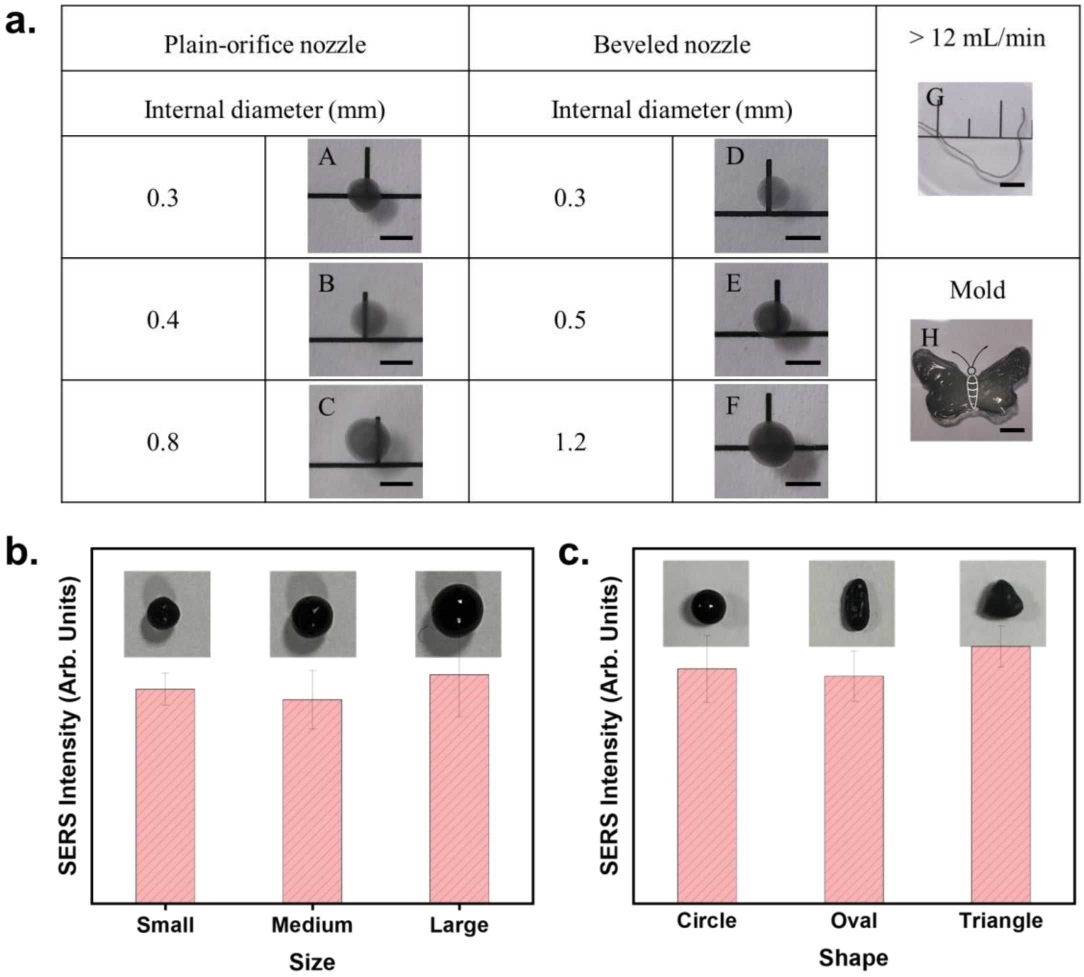

Figure S7. (a)Varied Shapes of the Plasmonic MXene Hydrogel. (A-F) Scale bar: 2 mm. (G) Scale bar: 4 mm. (H) Scale bar: 10 mm. SERS intensity (based on the peak intensity at 612 cm^{-1}) acquired by PM-Gel soaked in R6G solutions (10 nM) with (b) various sizes and (c) shapes.

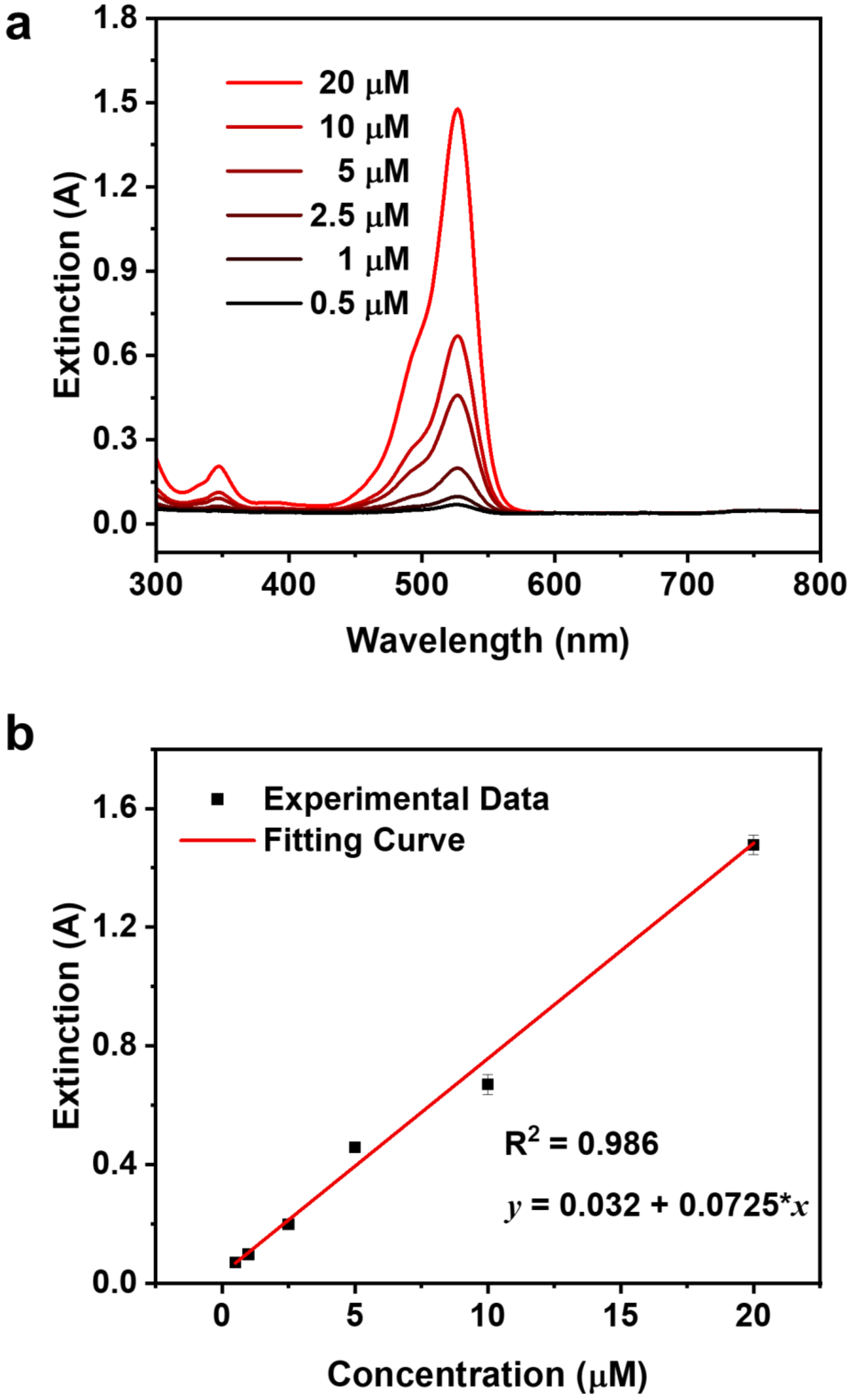

Figure S8. (a) Extinction spectra of R6G solutions of different concentrations. (b) The intensity of the extinction peak at 526 nm varies with concentration.

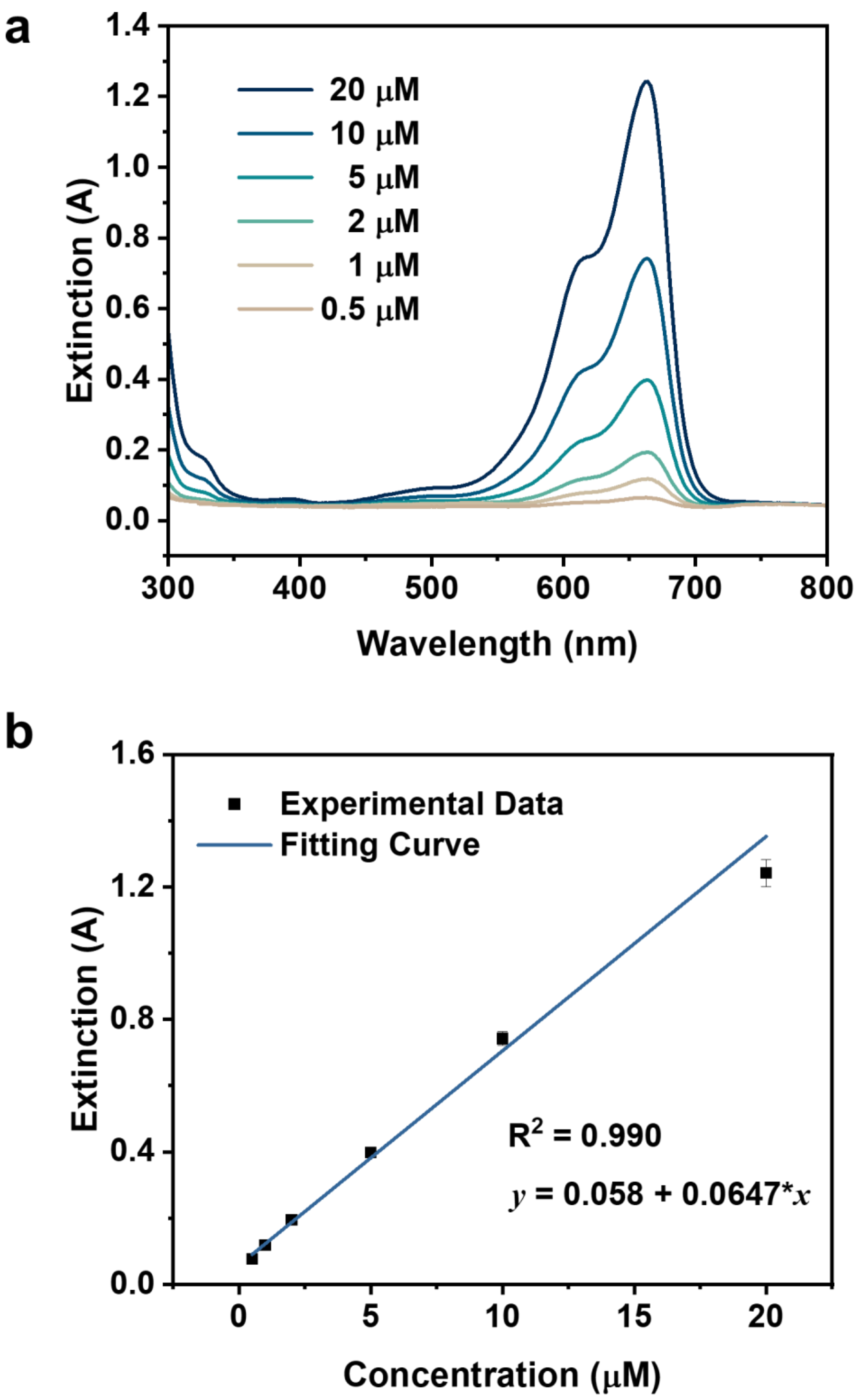

Figure S9. (a) Extinction spectra of MB solutions of different concentrations. (b) The intensity of the extinction peak at 663 nm varies with concentration.

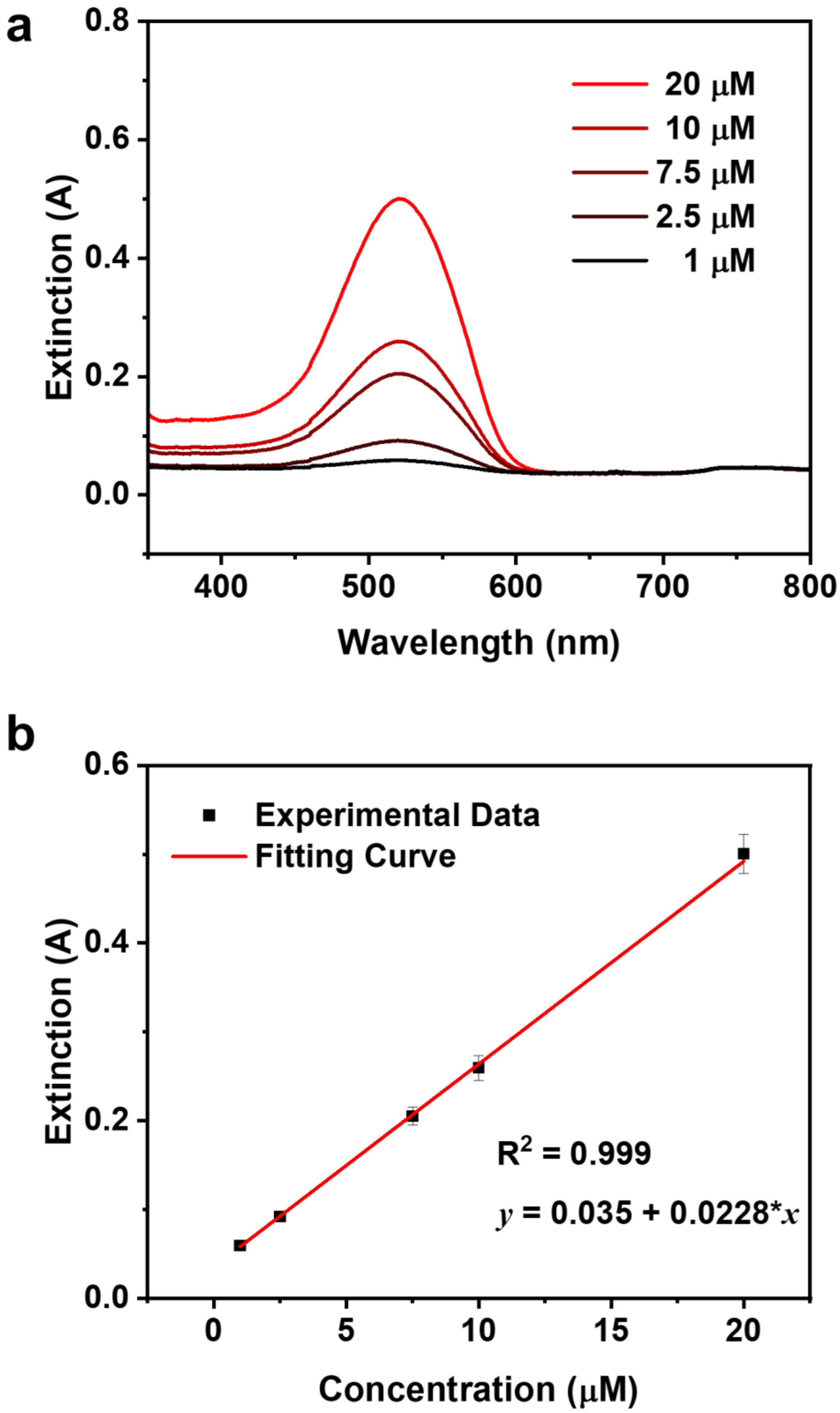

Figure S10. (a) Extinction spectra of AR solutions of different concentrations. (b) The intensity of the extinction peak at 522 nm varies with concentration.

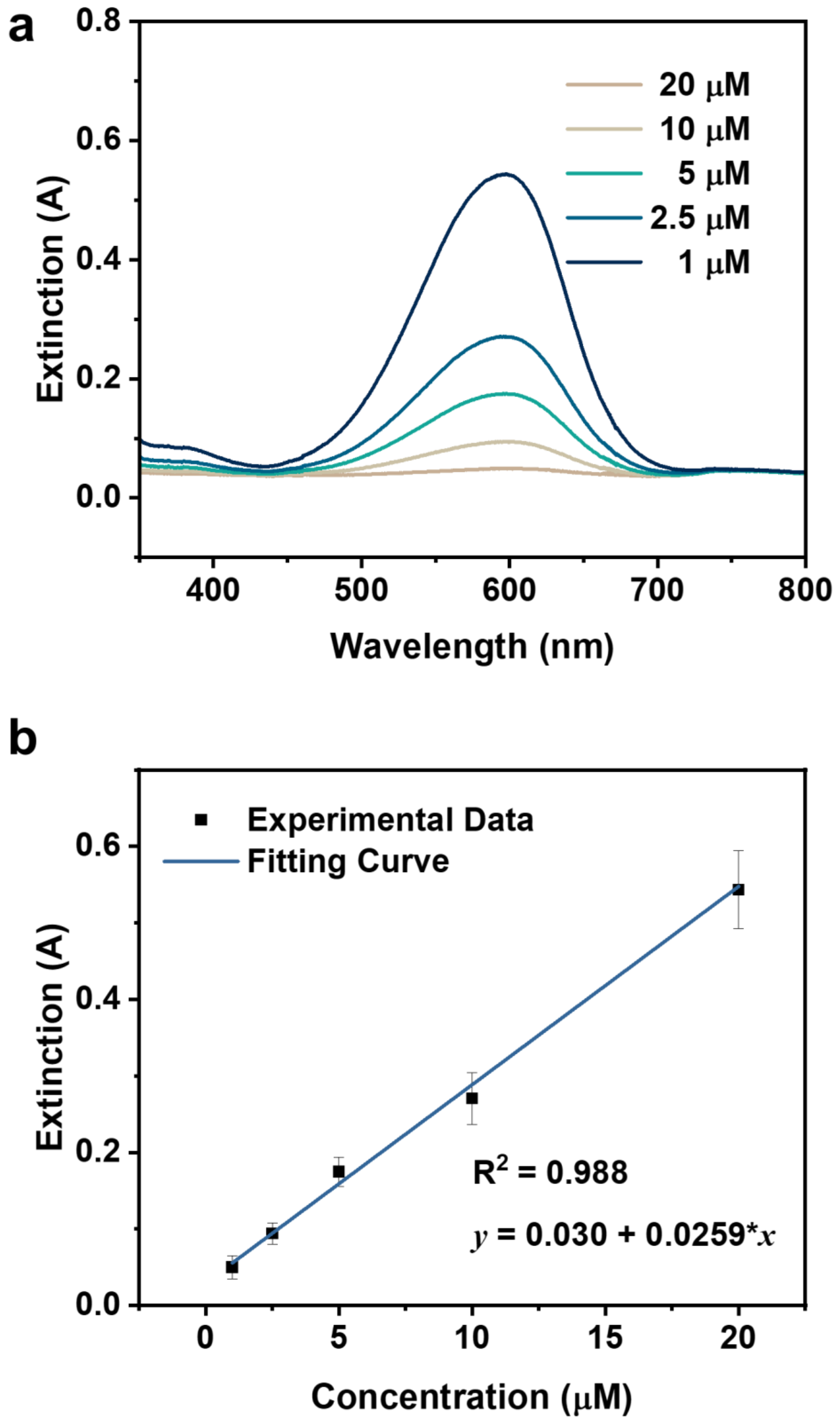

Figure S11. (a) Extinction spectra of AB solutions of different concentrations. (b) The intensity of the extinction peak at 596 nm varies with concentration.

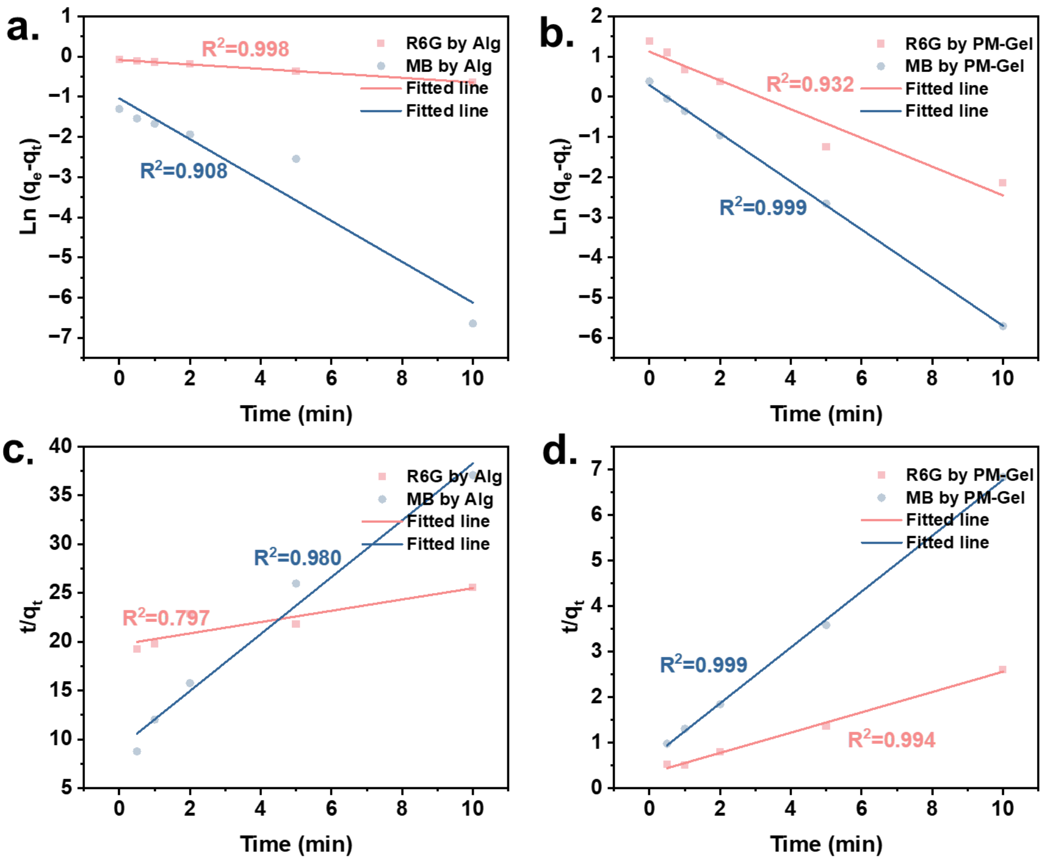

Figure S12. Kinetic modelling analysis for Alg and PM-Gel. (a) Pseudo-first-order model. (b) Pseudo-second-order model.

The corresponding kinetic equations are as follows.

For pseudo-first-order kinetic model:

$$ln(q_e - q_t) = ln\, q_e - K_1 t$$

For pseudo-second-order kinetic model:

$$\frac{t}{q_t} = \frac{1}{K_2 q_e^2} + \frac{t}{q_e}$$

Here, t is the reaction time, q_e and q_t are the adsorption amounts at equilibrium and reaction time, K_1 and K_2 are the rate constants for the pseudo-first-order and pseudo-second-order model.

The fitting coefficient R^2 value of the pseudo-second-order kinetic model is higher than that of the pseudo-first-order kinetic model (Figure S12, Table S13). Therefore, the main adsorption mechanism should be the chemical adsorption.

In addition, calculation, the saturation adsorption rate (q_e) of pure Alg hydrogel and

PM-Gel are 1.727 and 4.484 mg/g for R6G, respectively. The enhanced adsorption capacity is evidently dependent on the incorporation of MXene material. Considering the mass ratio of 421:1 between the PM-Gel and MXene (see experimental section), the q_e of $Ti_3C_2T_x$ MXene for R6G is calculated to be 1196.996 mg/g, as estimated below:

$$q_{e\_MXene\_R6G} = (q_{e\_PM-Gel} - q_{e_{SA}} \times \frac{400}{421}) \times 400$$

Similarly, $q_{e\_MXene\_MB}$ can be estimated as 548.934 mg/g.

Table S13. Overview of the Kinetic Parameters

Model	Parameter	R6G by Alg	MB by Alg	R6G by PM-Gel	MB by PM-Gel
Pseudo-first-order	q_e (mg/g)	0.919	0.271	3.967	1.467
	K_1 (min^{-1})	0.056	0.310	0.619	0.762
	R^2	0.998	0.908	0.932	0.999
Pseudo-second-order	q_e (mg/g)	1.727	0.343	4.484	1.630
	K_2 (min^{-1})	0.017	0.930	0.152	0.591
	R^2	0.797	0.980	0.994	0.999

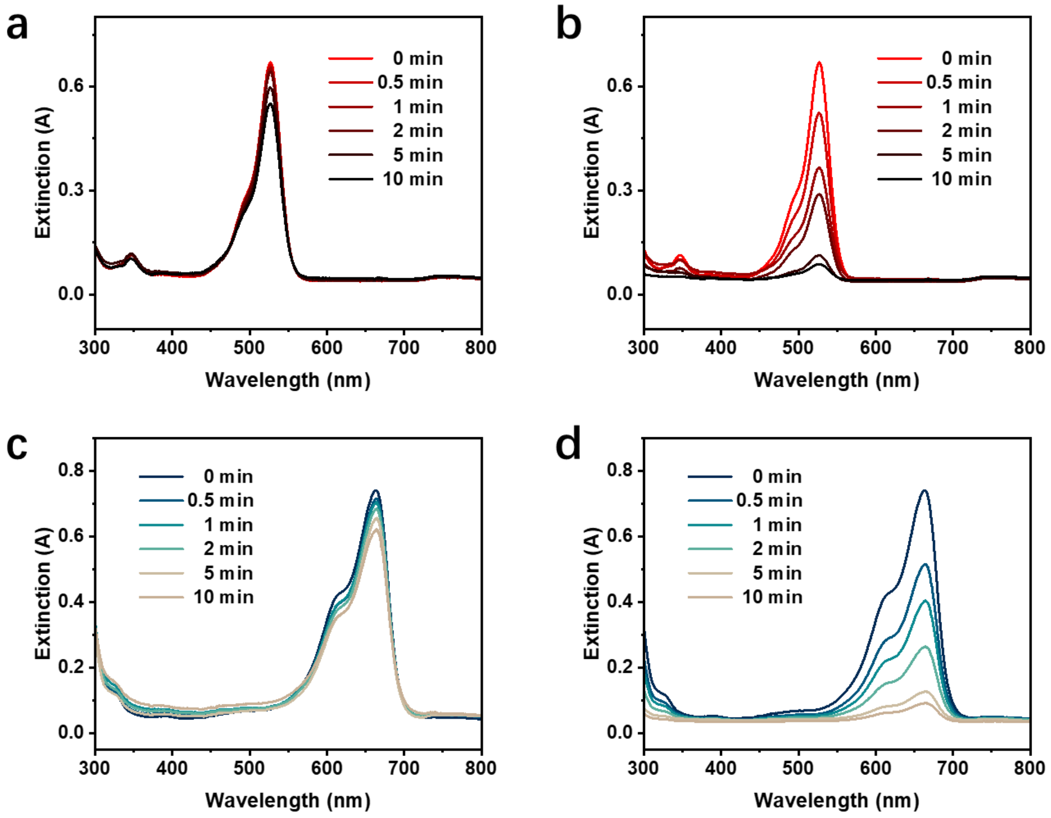

Figure S14. Extinction spectra of R6G solutions incubated with (a) pure alginate hydrogel and (b) MXene-doped microrobots for different time. Extinction spectra of MB solutions incubated with (c) pure alginate hydrogel and (d) MXene-doped microrobots for different time.

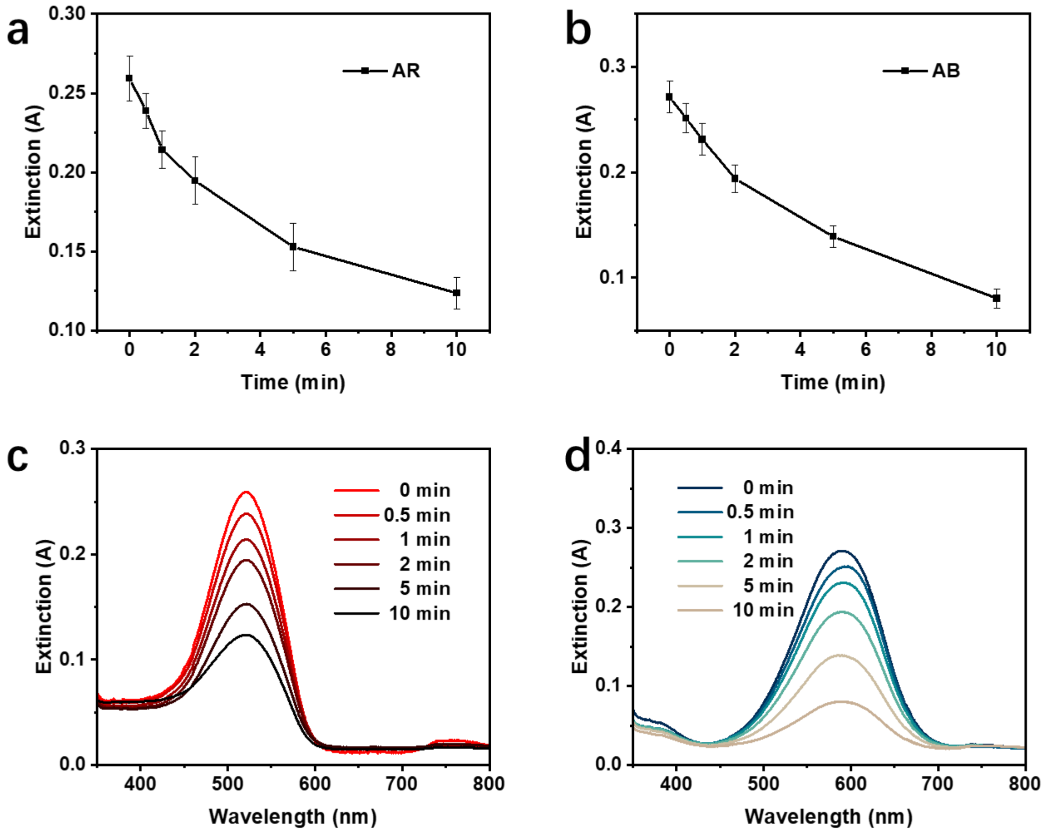

Figure S15. Extinction spectra of (a, c) AR or (b, d) AB solutions incubated with MXene-doped microrobots for different time.

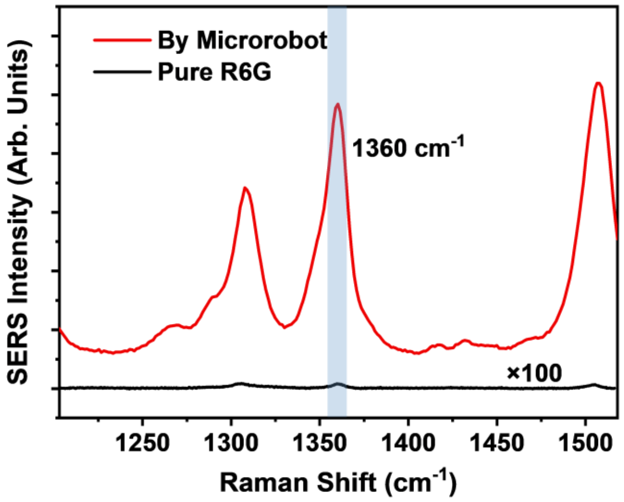

Figure S16. The spectra of R6G detected by different substrates.

The enhancement factor (EF) of the proposed substrate was calculated using R6G. The calculation of EF is demonstrated as follows.

According to the literatures (*J. Phys. Chem. C.* **2007**, 111, 13794-13803), EF is calculated by:

$$EF = \frac{N_{Raman}}{N_{SERS}} \times \frac{I_{SERS}}{I_{Raman}}$$

where, N_{Raman} and N_{SERS} are the number of R6G molecules in the illuminated spot for Raman and SERS detection, respectively. The intensity (I_{SERS}, I_{Raman}) is the SERS and Raman intensity at 1360 cm^{-1} peak normalized by acquisition time, respectively.

In the past experiments (*ACS Nano* **2021**, 15, 12996–13006), we have obtained the data of N_{Raman}, which was calculated to be 5.6×10^9.

N_{SERS} for MXene-loaded substrate can be estimated as

$$N_{SERS} = \frac{A_{spot}}{A_{microrobot}} \times c_{R6G} \times N_A \times V_{Solution} \times \eta$$

where, A_{spot} is the surface area of laser spot (diameter ~ 3 μm), $A_{microrobot}$ is the volume of microrobot (diameter ~ 2 mm), c_{R6G} is the concentration of the solution (0.01 μM), $V_{Solution}$ is the volume of the solution (2 mL), while η is the adsorption rate. Here, η was identified as 20%. So N_{SERS} is estimated to be 5.4×10^6.

On the other hand, according to Raman and SERS spectra, I_{Raman} is 9.4, I_{SERS} is 50000. So, the analytical EF can be calculated as 5.5×10^6.

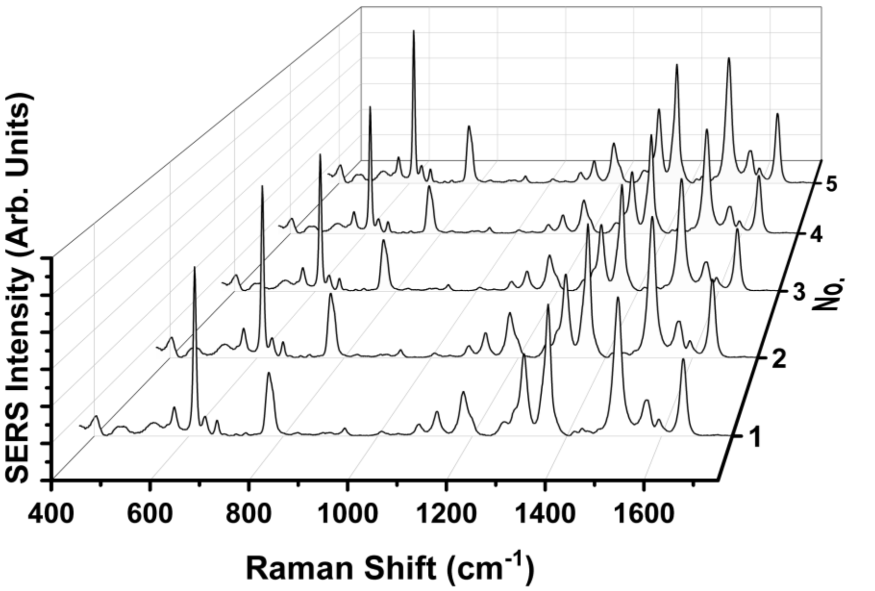

Figure S17. The spectra of R6G (1 μM) detected by different microrobots.

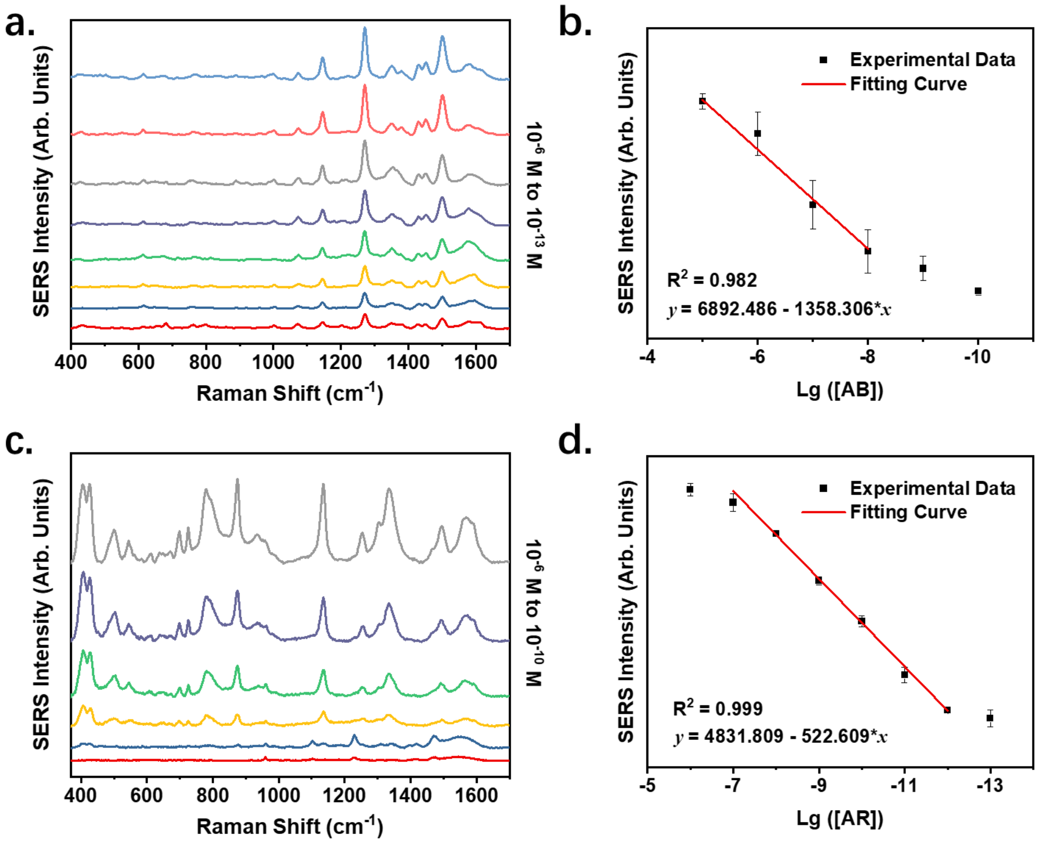

Figure S18. Investigation of the SERS activity of the microrobot for anionic dyes. Detection results of (a) AR, and (c) AB at different concentrations by the microrobot. Before tests, the microrobots were immersed in the dye solutions at 10 μM for 2 hours and then washed by pure water. SERS intensity of (b) AR, and (d) AB at different concentrations based on the peak at 1270 or 1168 cm^{-1}, respectively.

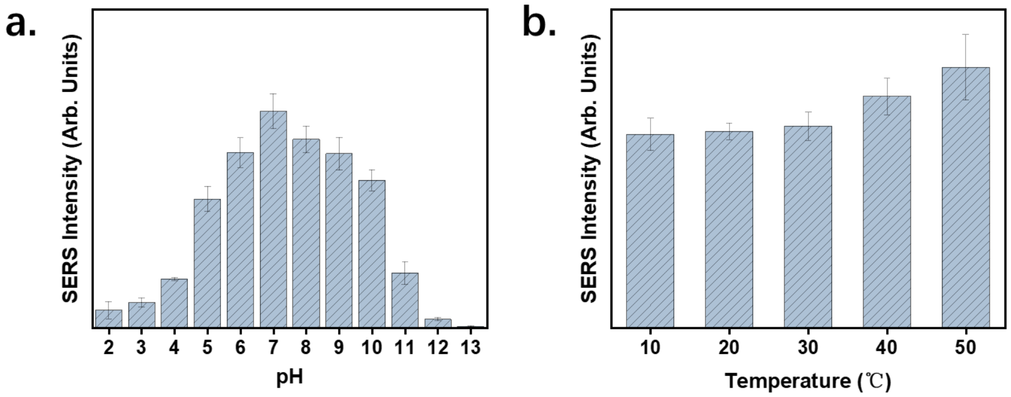

Figure S19. SERS intensity acquired by the PM-Gel soaked in R6G solutions (10 μM) with (a) various pH values and (b) different temperature.

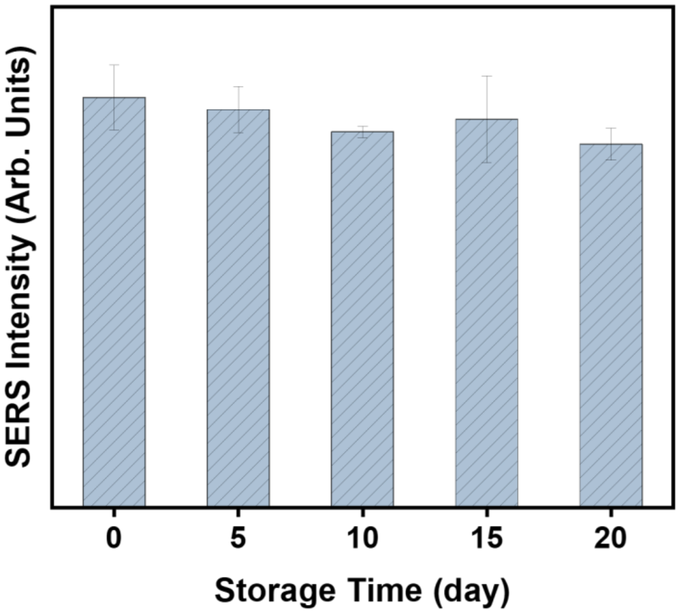

Figure S20. Changes in sensing performance of the PM-Gel after a 20-day storage at 20°C in air. The detection targets are all R6G solutions of 10 μM.

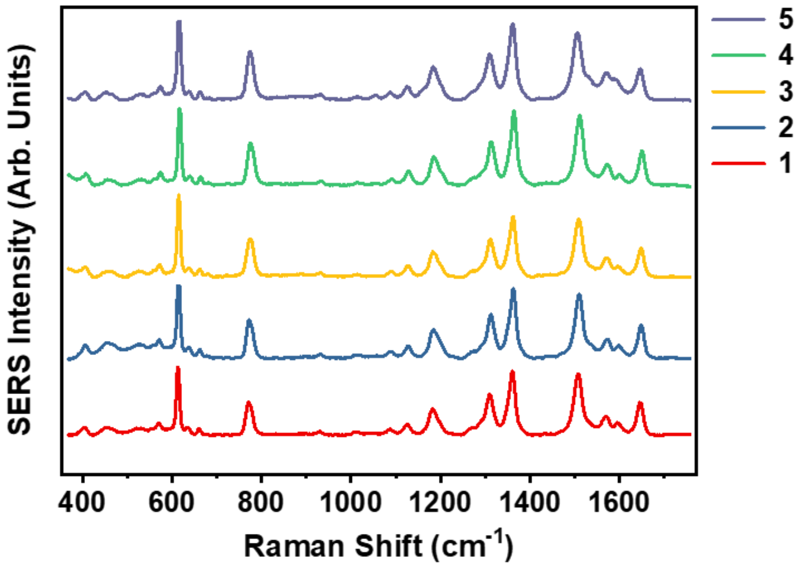

Figure S21. Reusability of the PM-Gel for R6G dye adsorption (10 μM). Before each assay, a 1-hour soak in ethanol was used to reset the hydrogel.

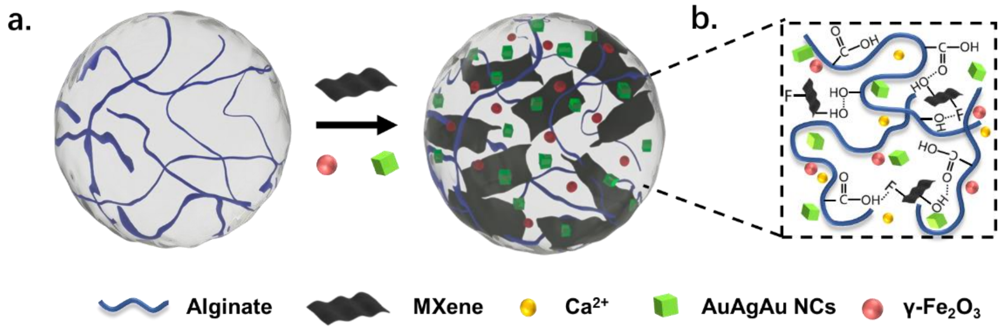

Figure S22. (a) The structure diagram of a spherical robot powered by plasmonic MXene hydrogel containing γ-Fe_2O_3 nanoparticles. (b) The schematic diagram of the cross-linked structure of the proposed hydrogel containing γ-Fe_2O_3 nanoparticles.

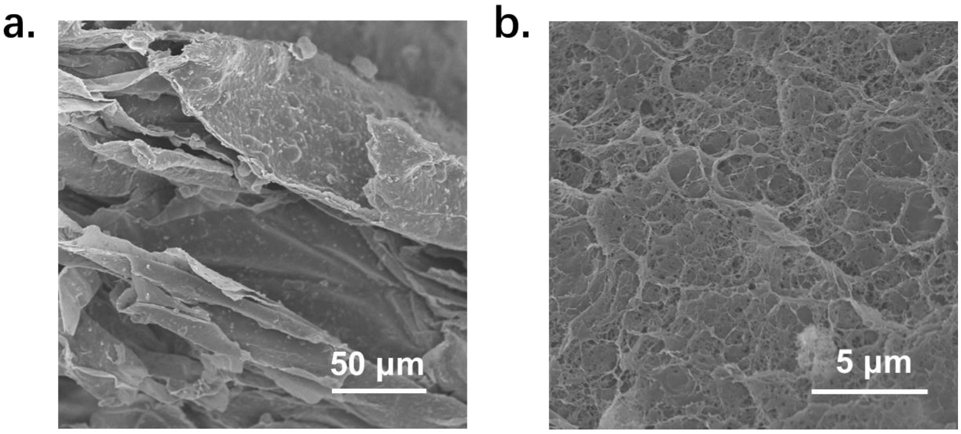

Figure S23. The typical SEM images of the freeze-dried spherical microrobot.

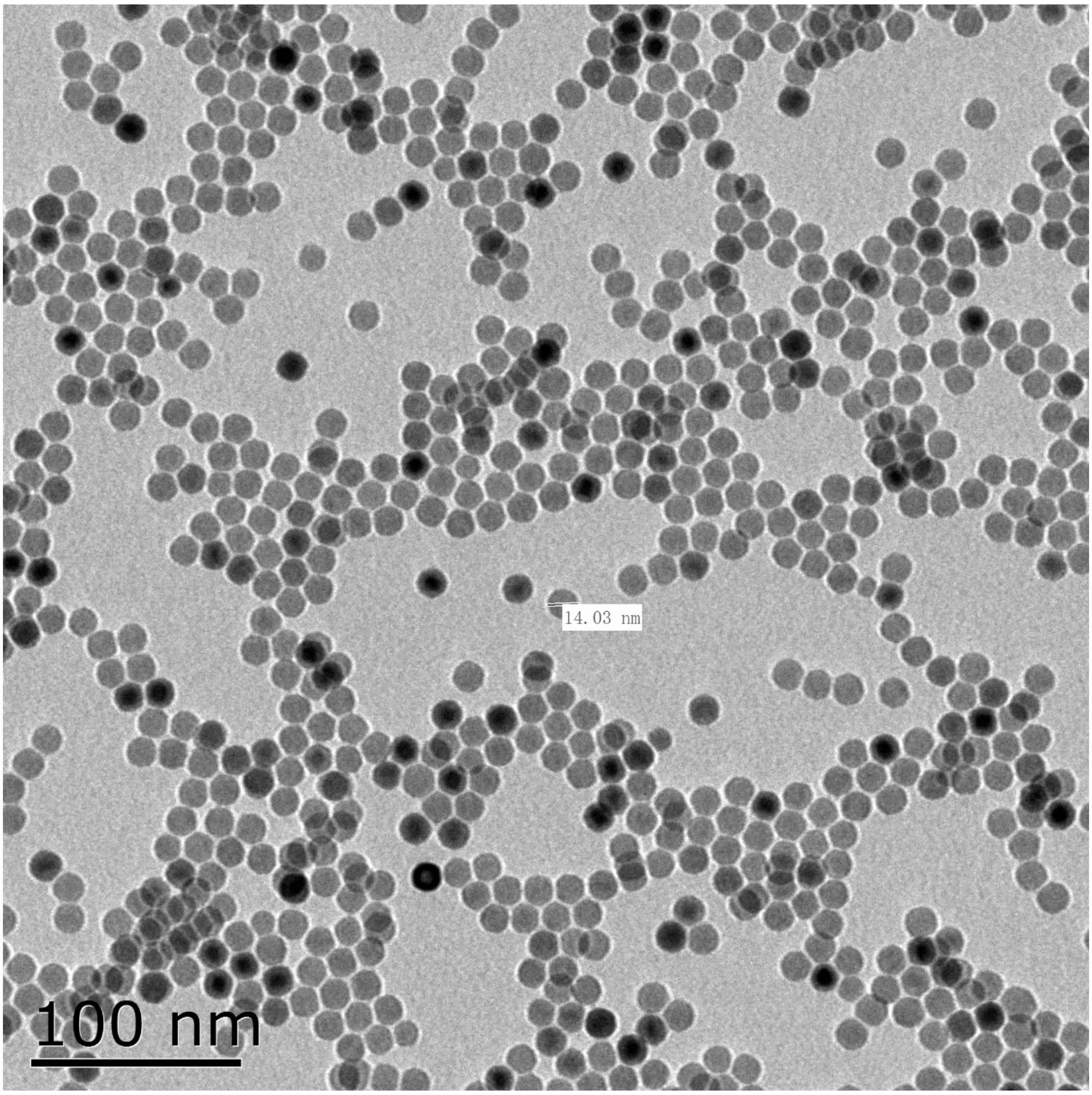

Figure S24. The TEM image of γ-Fe_2O_3 nanoparticles.

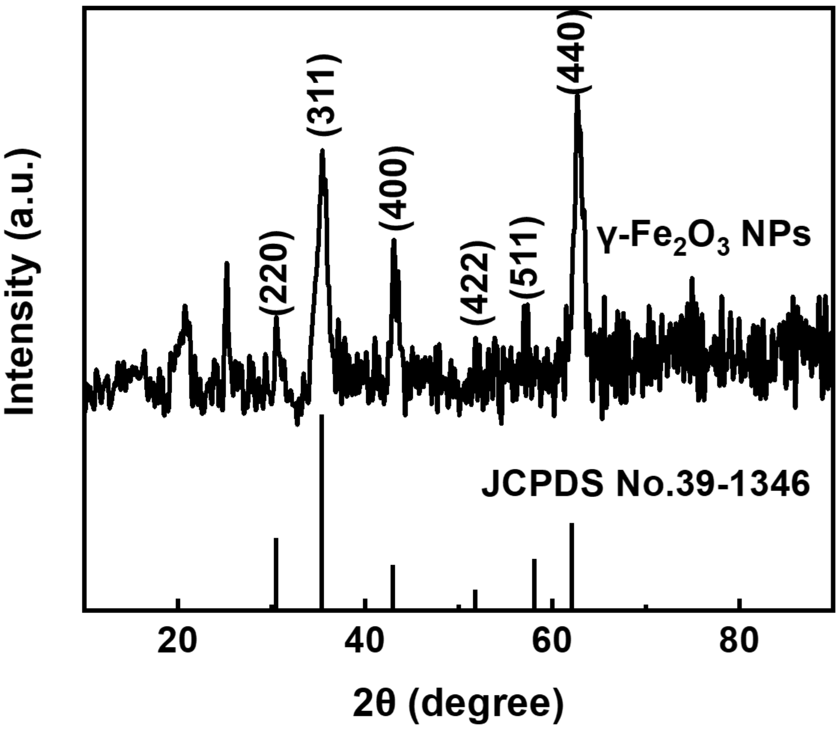

Figure S25. The XRD spectra of the γ-Fe_2O_3 nanoparticle and standard γ-Fe_2O_3 (JCPDS No. 39-1346). The XRD pattern of the γ-Fe2O3 reveals that the diffraction peaks are located at 2θ of 30.24°, 35.63°, 37.24°, 43.28°, 53.73°, 57.27°, and 62.92°. These peaks correspond to the diffraction of (111), (220), (311), (222), (400), (422), (511), and (440) crystal faces, respectively.

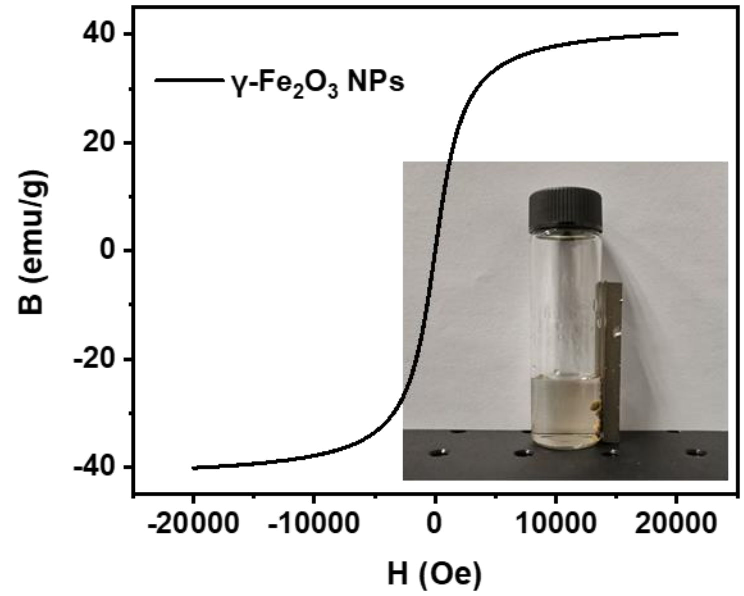

Figure S26. The hysteresis curves of γ-Fe_2O_3 nanoparticles. The inset photo is the PM-Gel-based microrobots dragged aside by a magnet.

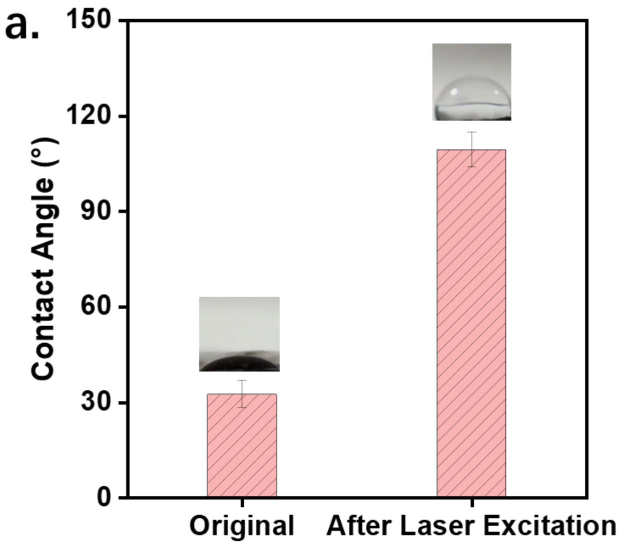

Figure S27. The changes in contact angles of the PM-Gel. The power density of the light is 3 W/cm^2.

Table S28. Overall features of our work in comparison with other typical materials.

Materials	Target pollutants	Removal percentage	Maximum adsorption capacity	Sensitivity (Limit of detection)	Versatility	Reusability	References
COF-CNT	Malachite Green (MG) and Methyl Orange (MO)	97%	553.55 and 226.12 mg/g	N/A	N/A	Yes	*Environ. Chem. Eng.* **2023**, 11(3), 60-75.
$CoFe_2O_4$/GO	MB, rhodamine B (RhB) and MO	96.8%	355.9, 284.9 and 53.0 mg/g	N/A	N/A	Yes	*Sep. Purif. Technol.* **2020**, 238, 116400.
Magnetic Chitosan Corn Straw Biochar (MCB)	AR	94.5-98.6%	404.18 mg/g	N/A	N/A	Yes	*Int. J. Bio.l Macromol. I.* **2022**, 199, 234-242.
CTAB/MXene/BAC (CMAC)	Allure red (AR), congo red (CR) and sunset yellow (SY)		AR: 311.85 mg/g<br>CR: 1714.67 mg/g	N/A	N/A	Yes	*Chem. Eng. J.* **2023**, 451 (3), 138735.
Ag@βCD NPs-alginate hydrogel bubbles	MB	90%		10 nM	Detection and photocatalytic	No	*Environ Sci-nano*. **2020**, 7(12), 3888-3900.
Magnetic hydrogels containing activated carbon	Cr (VI)	93%	0.76 mg/g	N/A	N/A	Yes	*Environ. Sci.: Water Res. Technol., **2024**, 10, 551*
Ch-PEDOT:PSS	MO and MB	MO:66%<br>MB:100%		10 μM	N/A	Yes	*Gels*. 2024, 10(1), 37.
PM-Gel	R6G, MB, AR and AB	95%	R6G: 1197.00 mg/g	~3.8 aM	Remote navigation, rapid removal, and molecular identification	Yes	This work

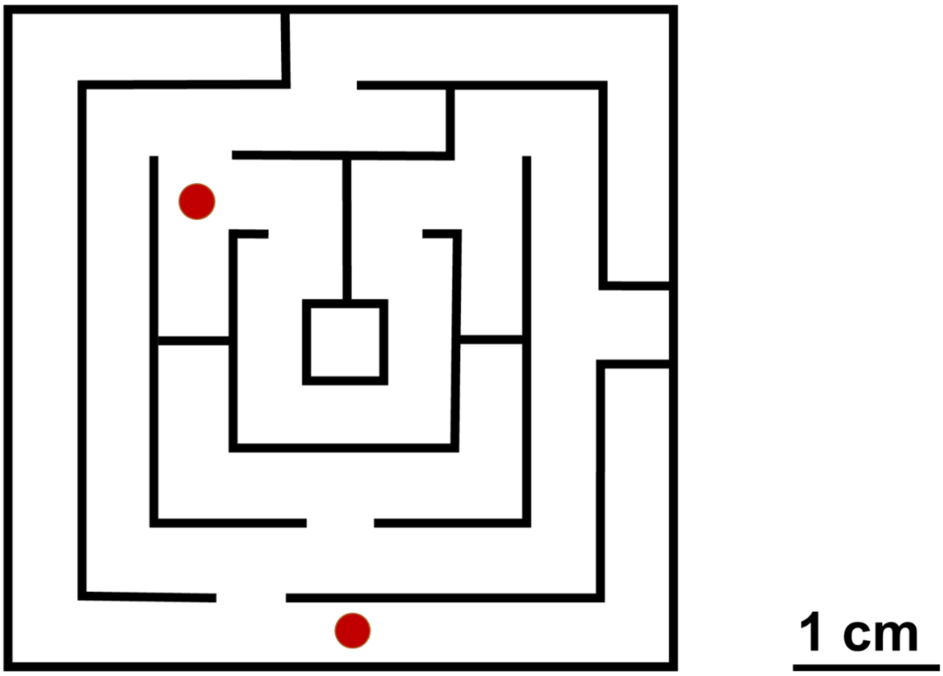

Figure S29. The plane view of the maze-like microfluidic channels. The red labels mark the "Start" and "Target" points in the proof-of-concept experiment.
